# TGS-TB: Total Genotyping Solution for *Mycobacterium tuberculosis* Using Short-Read Whole-Genome Sequencing

**DOI:** 10.1371/journal.pone.0142951

**Published:** 2015-11-13

**Authors:** Tsuyoshi Sekizuka, Akifumi Yamashita, Yoshiro Murase, Tomotada Iwamoto, Satoshi Mitarai, Seiya Kato, Makoto Kuroda

**Affiliations:** 1 Pathogen Genomics Center, National Institute of Infectious Diseases, Shinjyuku-ku, Tokyo, Japan; 2 Molecular Epidemiology Division, The Research Institute of Tuberculosis/Japan Anti-Tuberculosis Association, Kiyose, Tokyo, Japan; 3 Department of Infectious Diseases, Kobe Institute of Health, Chuo-ku, Kobe, Japan; 4 Bacteriology Division, Department of Mycobacterium Reference and Research, Research Institute of Tuberculosis, Japan Anti-Tuberculosis Association, Kiyose, Tokyo, Japan; 5 Research Institute of Tuberculosis, Japan Anti-Tuberculosis Association, Kiyose, Tokyo, Japan; University of Hyderabad, INDIA

## Abstract

Whole-genome sequencing (WGS) with next-generation DNA sequencing (NGS) is an increasingly accessible and affordable method for genotyping hundreds of *Mycobacterium tuberculosis* (Mtb) isolates, leading to more effective epidemiological studies involving single nucleotide variations (SNVs) in core genomic sequences based on molecular evolution. We developed an all-in-one web-based tool for genotyping Mtb, referred to as the Total Genotyping Solution for TB (TGS-TB), to facilitate multiple genotyping platforms using NGS for spoligotyping and the detection of phylogenies with core genomic SNVs, IS*6110* insertion sites, and 43 customized loci for variable number tandem repeat (VNTR) through a user-friendly, simple click interface. This methodology is implemented with a KvarQ script to predict MTBC lineages/sublineages and potential antimicrobial resistance. Seven Mtb isolates (JP01 to JP07) in this study showing the same VNTR profile were accurately discriminated through median-joining network analysis using SNVs unique to those isolates. An additional IS*6110* insertion was detected in one of those isolates as supportive genetic information in addition to core genomic SNVs. The results of in silico analyses using TGS-TB are consistent with those obtained using conventional molecular genotyping methods, suggesting that NGS short reads could provide multiple genotypes to discriminate multiple strains of Mtb, although longer NGS reads (≥300-mer) will be required for full genotyping on the TGS-TB web site. Most available short reads (~100-mer) can be utilized to discriminate the isolates based on the core genome phylogeny. TGS-TB provides a more accurate and discriminative strain typing for clinical and epidemiological investigations; NGS strain typing offers a total genotyping solution for Mtb outbreak and surveillance. TGS-TB web site: https://gph.niid.go.jp/tgs-tb/.

## Introduction

An estimated 8.6 million people developed tuberculosis (TB) in 2012, and 1.3 million individuals died from this disease. WHO reported 450,000 new cases of multidrug resistant (MDR) *Mycobacterium tuberculosis* (Mtb) resistant to at least isoniazid and rifampicin worldwide [1]. Molecular genotyping of Mtb has been well developed [2]; three main typing methods, specifically IS*6110* restriction fragment length polymorphism (RFLP), spoligotyping (spacer oligonucleotide typing), and variable-number tandem repeat (VNTR) analysis, are currently used for fingerprinting Mtb strains to detect recent transmission.

IS*6110*-RFLP typing is a standard genotyping approach [3], but requires subculturing the isolates for several weeks to obtain sufficient DNA, and the typing procedure distinguishes a limited number of polymorphisms. Mtb contains 10 to 50 copies of a 36-bp direct repeat (DR) in clustered regularly interspaced palindromic repeats (CRISPRs), and the spacer sequences between DRs have different nucleotide sequences among strains. Thus, the pattern of spacers in a strain can be used for spoligotyping [4]. However, spoligotyping has less resolving power to discriminate among Mtb strains compared with IS*6110* genotyping [5]. The Mtb genome contains many mycobacterial interspersed repeat units (MIRUs) and MIRU-VNTR. MIRU-VNTR typing has progressed and is currently used to visualize the transmission of multiple Mtb strains, yielding intrinsically digital results that can be easily catalogued in a computer database [6]. Among more than 40 VNTR loci on the Mtb chromosome, MIRU-VNTR 15 and 24 loci have been proposed as the international standard [7]. However, the discriminatory power of this technique is not sufficient in countries such as East Asia and Russia with a high proportion of Beijing-type Mtb. Lineage- or sublineage-specific loci should be additionally investigated to increase the discriminative power of genotyping [8].

Genetic elements for molecular epidemiological genotyping techniques do provide adequate discriminatory power for distinguishing *M*. *tuberculosis* strains. However, the clustered strains defined using these methods might be distantly related, both genetically and historically, reflecting the low reliability of these tests to distinguish recent from past transmissions [9]. Thus, epidemiological investigations are typically needed to confirm recent transmission and remote infection.

Whole-genome sequencing (WGS) using next-generation DNA sequencing (NGS) has emerged as an increasingly accessible and affordable method for genotyping hundreds of Mtb isolates, leading to more effective epidemiological studies involving single nucleotide polymorphisms (SNPs) in the core genomic sequence based on the molecular evolutionary clock [9–14]. Genome-based clustering patterns are more consistent with contact tracing data and the geographical distribution of the cases compared with clustering patterns based on classical genotyping [15]. WGS facilitates the effective tracing of the Mtb complex (MTBC). Niemann et al. demonstrated that WGS revealed genomic heterogeneity among drug-susceptible and drug-resistant Mtb isolates with identical IS*6110* fingerprints and 23 out of 24 MIRU-VNTR loci [16]. Such heterogeneity is not detected using conventional MTBC genotyping, and some aspects of Mtb transmission dynamics could be missed or misinterpreted. When the overall genetic diversity of circulating clones is restricted, standard genotyping might not distinguish between relapse and exogenous re-infection. Bryant et al. demonstrated that WGS facilitates the differentiation of relapse and re-infection cases, with higher resolution through small (0 to 6 SNPs) and large (1,306 to 1,419 SNPs) distances [13]. It has been suggested that the mutation rate is constant at approximately 0.5 single nucleotide polymorphisms per genome per year in latent, active and re-activated diseases [9, 13, 17, 18]. Walker et al. established that most Mtb isolates were within five SNPs on the genome of another isolate obtained from the same individual or from a household contact [9].

Freely accessible web services facilitate the genotyping of isolated strains alone or in comparison with reference strains from major MTBC lineages. Currently, MIRU-VNTRplus web tools (http://www.miru-vntrplus.org) are available for analyzing MLVA data (MtbC15-9 type) in combination with other complementary typing data, including spoligotypes, regions of difference (RDs), SNPs in antimicrobial target genes and susceptibility information [19]. In addition to conventional genotyping tools, Kohl et al. recently provided core genomic multilocus sequence typing (cgMLST) tools to expand the approach for standardized WGS-based genotyping [20]. *In silico* spoligotyping is available using SpolPred to more accurately and rapidly determine a spoligotype [21]. The stand-alone, user friendly tool KvarQ has been reported to assign MTBC lineage/sublineages, SNP-barcodes and potential antimicrobial resistance (AMR) genes within 2 minutes based on SNP analysis directly from NGS short reads [22]. Similarly, PhyResSE also facilitates the lineage genotyping and AMR detection of Mtb using web-based tools [23]. The comprehensive genomic variation map for Mtb is available on the PolyTB web-based tool (http://pathogenseq.lshtm.ac.uk/polytb) to visualize the resulting MTBC genetic variations (74,039 SNPs, 4820 indels and 800 deletion sites) and important meta-data (e.g., *in silico* inferred strain-types and locations) at a genome and global scale [24]. Unexpectedly, newly obtained raw sequences cannot be analyzed using PolyTB, and conventional typing data are not available on the PolyTB resource. PhyTB illuminates *M*. *tuberculosis* genomic variation within epidemiological, geographical and phylogenic settings from 1,601 *M*. *tuberculosis* isolates, facilitating the assessment of genotype-phenotype associations [25].

WGS-based genotyping (cgMLST) [20], SpolPred [21], KvarQ [22] and PhyResSE [23] provide partial *in silico* genotyping analysis but do not include other conventional genotyping formats, such as IS*6110*-RFLP and MIRU-VNTR. This independent genotyping tool has not been integrated into one system. Thus, it can be laborious to determine each genotype through multiple genotyping programs.

**Fig 1:**
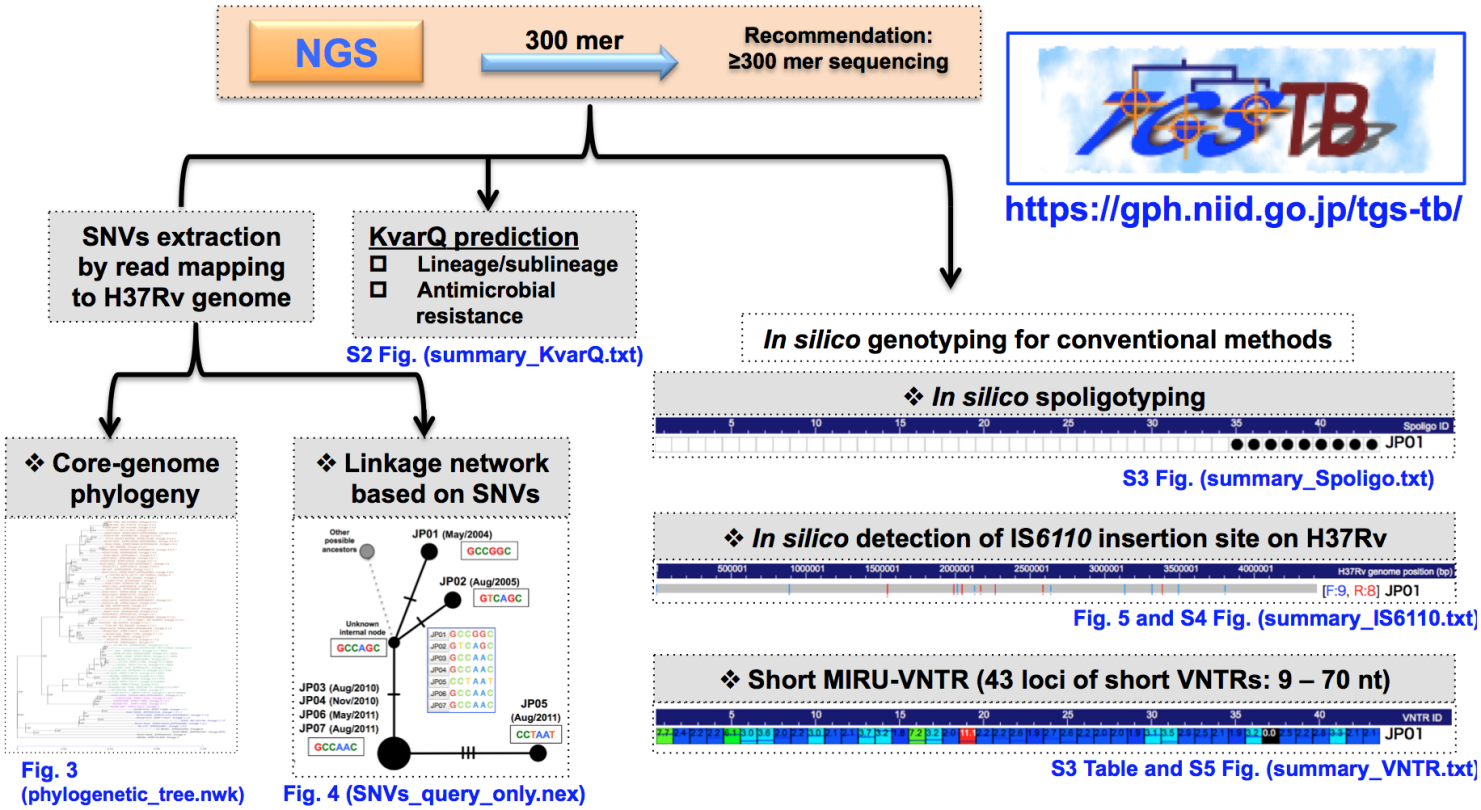
Schematic representation of the TGS-TB system.

Here, we present a Total Genotyping Solution for TB (TGS-TB) web-based tool to facilitate multiple genotyping formats using NGS for the analysis of phylogenies with core genomic single nucleotide variations (SNVs), linkage network analysis of outbreak strains, spoligotyping, the analysis of IS*6110* insertion sites and VNTRs (our customized short TR on 43 loci) through a user-friendly, simple click interface (Fig 1). The prediction of MTBC lineages/sublineages and potential AMRs based on the KvarQ script [22] is also included in TGS-TB web tools. Multiple NGS data obtained from outbreak strains can be accepted through TGS-TB, and the discrimination of strain-specific genotypes can be elucidated to investigate outbreaks, thereby contributing to TB surveillance.

## Results and Discussion

Although WGS provides an adequate solution for molecular epidemiology, traditional genotyping methods are still used effectively. We have developed an all-in-one bioinformatics tool to implement both traditional and newly developed techniques (Fig 1). MiSeq is one of the best sequencers to accomplish this project, as MiSeq provides sufficient accuracy to assign SNVs and AMR-related genetic alterations and longer read lengths, up to 300-mer, to assign spoligotypes, IS*6110* insertion sites and sMIRU-VNTRs. Indeed, 350-mer (read1) x 250-mer (read2) paired-end sequencing resulted in 70% of 300-mer nucleotide sequences with Phred quality scores above 30 (S1 Fig), indicating sufficient accuracy for multiple *in silico* genotypings.

### Basic function of TGS-TB

**Fig 2:**
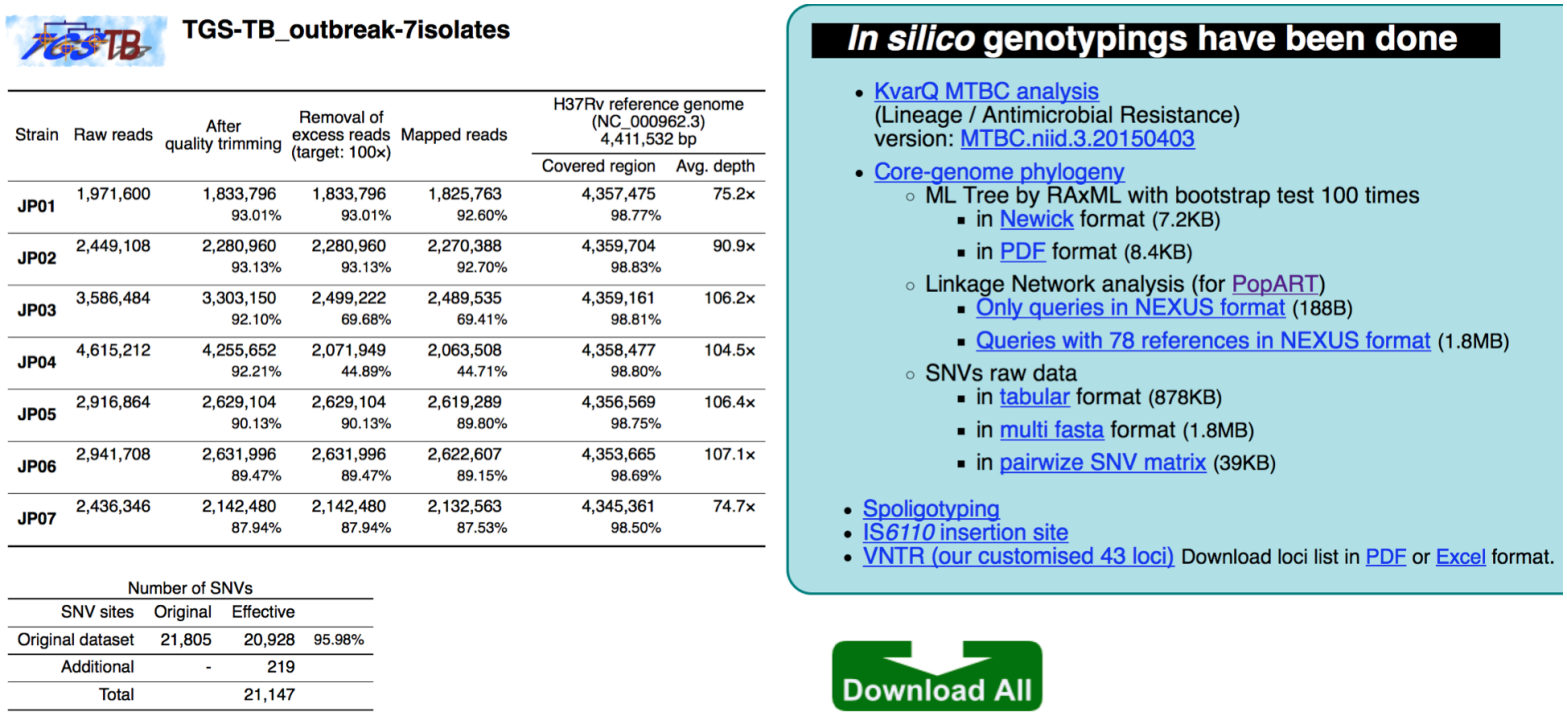
Sample results obtained from TGS-TB. The NGS reads of seven Mtb isolates were investigated, and the resulting basic information, such as number of trimmed map reads and the coverage region-depth, is shown. In total, 21,805 core-genome SNVs are available in the TGS-TB; 20,928 (95.98%) SNVs are characterized, and 219 additional strain-specific SNVs sites can be implemented in the original dataset. The respective results for lineage, AMR, core genome phylogenetic tree (maximum-likelihood method with x100 bootstrapping), spoligotyping, IS*6110* insertion and sMIRU-VNTR typing can be viewed in a new window tab and retrieved using the “download all” button. The KvarQ script predicts the lineages/sublineages and AMRs, and the sample queries are assigned as a lineage 2/Beijing sublineage without AMRs (S2 Fig). The AMR target list in the original KvarQ (v2.0) program has been improved with the addition of more reliable genetic alterations for the *embA*, *gyrA*, *katG*, *pncA*, *rpoB*, *rpsL*, *rrs* and *inhA* genes (S1 Text).

Paired-end fastq.gz files can be uploaded to the TGS-TB web page, and an e-mail announcement will be sent to users when all analyses are completed. The resulting basic information, such as number of trimmed map reads and the coverage region depth, is reported for all tested isolates (Fig 2). The respective results for lineage, AMR, core genome phylogeny (maximum-likelihood method), linkage network of outbreak strains, IS*6110* insertion, spoligotyping and sMIRU-VNTR typing can be viewed in a new window tab and can also be retrieved using the “download all” button.

### Core-genome phylogeny

To further characterize Mtb lineage analysis comprehensively based on core genome phylogeny, complete/draft genome sequences and short read archives for 78 isolates (S1 Table) were selected from ~2,400 public available short read archives (SRA) of Mtb strains.

**Fig 3:**
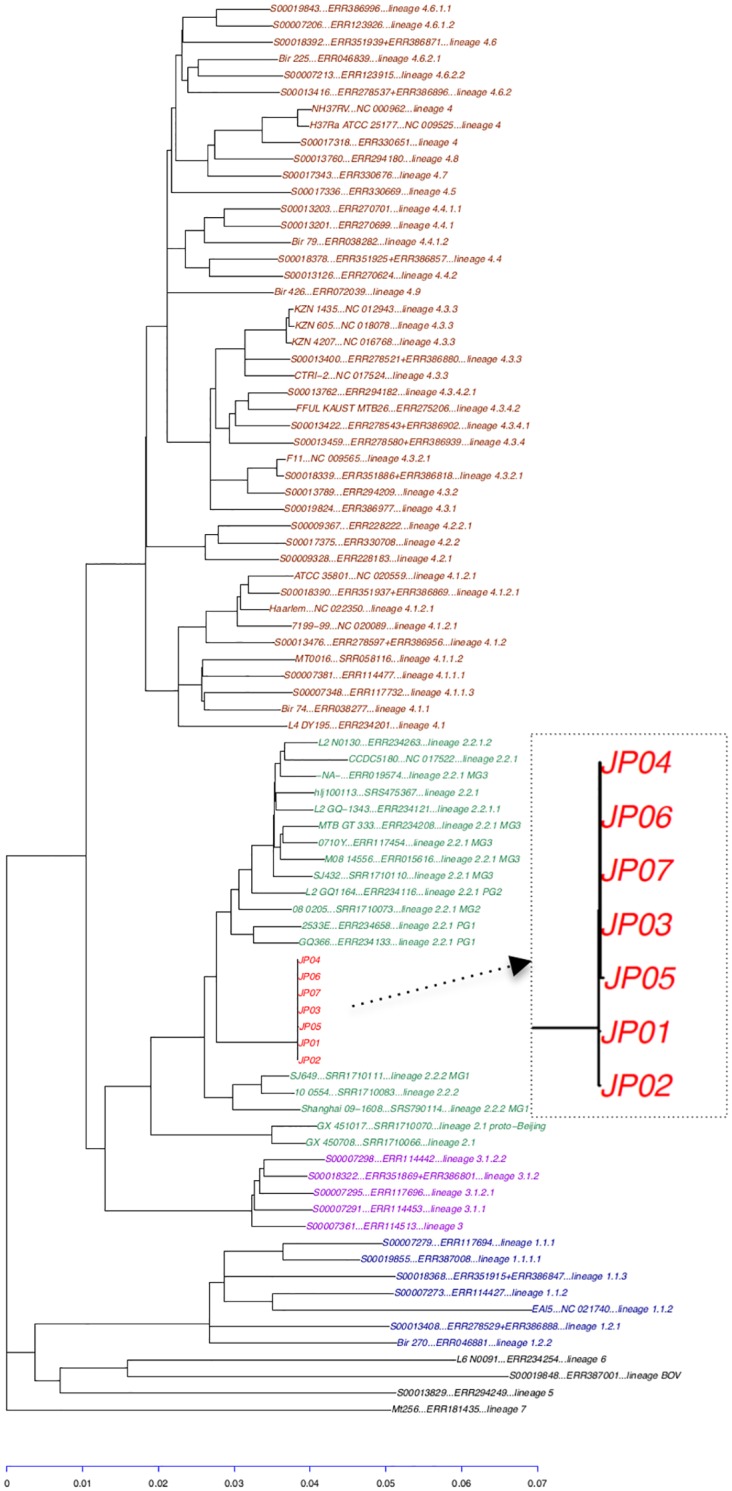
The core genome phylogeny obtained by the maximum-likelihood method with x100 bootstrapping.

In total, 21,805 core genome SNVs on the non-repetitive regions (S2 Table) are available in the TGS-TB, and 20,928 (95.98%) SNVs for the seven isolates (JP01 to JP07) were correctly extracted in the sample test (Fig 2). Additional query-specific novel SNVs can be identified in TGS-TB, and 219 additional strain-specific SNV sites can be implemented in the original dataset (21,805 core genome SNVs). A maximum-likelihood core genome phylogenetic tree is constructed based on the whole SNV dataset, including newly identified SNVs sites (Fig 3). The data for the original and query-specific SNVs can be downloaded as a tab-delimited file (summary_SNVs.txt) or fasta file (phylocoreGenome.fasta) for further phylogenetic analysis using more bootstrapping analyses or a Bayesian approach.

### Linkage network analysis using SNVs

**Fig 4:**
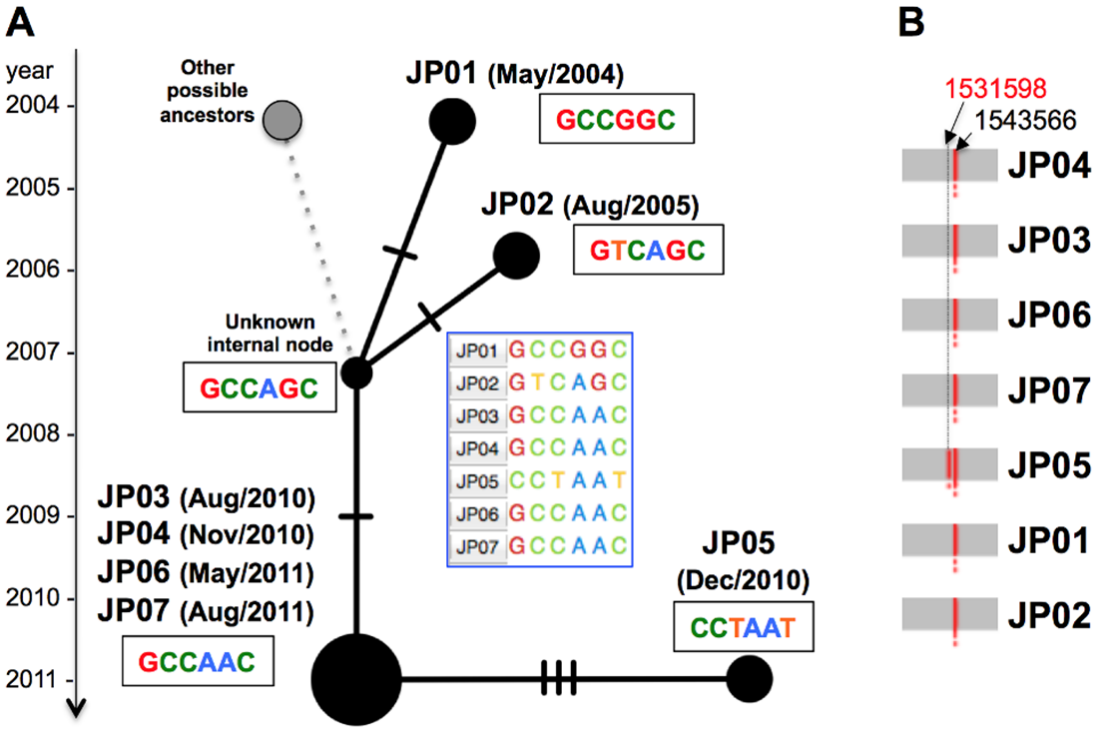
Median-joining network of the seven outbreak isolates based on the detected core genomic variations. A) The variations are summarized as nexus format files (.nex), and PopART visualizes the epidemiological linkages among the isolates through a user specified network method. The bars on the edge indicate the number of SNVs between the nodes (isolates). B) In addition to three SNV differences between JP05 and the outbreak isolates (JP03, JP04, JP06, and JP07), an additional IS*6110* insertion was detected at the 1,531,598 nt genome position in JP05, suggesting that JP05 could be unrelated to the outbreak, although the VNTR profile is consistent.

**Fig 5:**
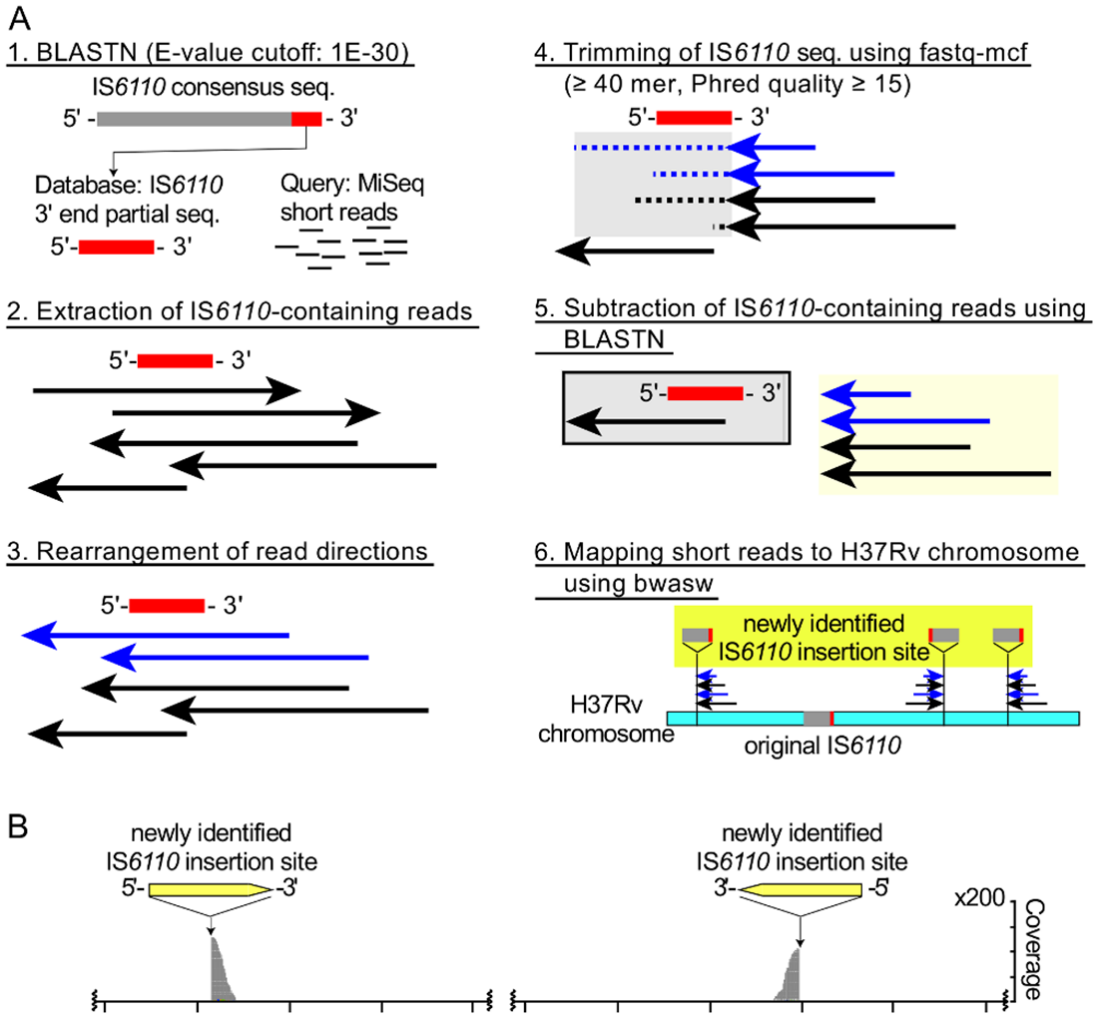
Schematic representation of the IS*6110* insertion detection strategy. A) The IS*6110* sequence (Acc.# X94955 and X94956)-positive short reads are collected (A1-2), rearranged (A3), trimmed (A4), subtracted (A5) and mapped to the Mtb H37Rv chromosome (NC_000962.3) [30] using BWA-SW mapping [29]. B) Typical read mapping profile for the detection of the IS*6110* insertion site in both directions.

Although the core genome phylogeny database includes 78 references, it is too computationally intensive to determine the precise differences for only outbreak-related query isolates. Thus, query-specific SNV sites are simple datasets used to perform further epidemiological investigations. Among the tested seven isolates (four from the outbreak and three from related isolates with similar MIRU-VNTR loci [26]), 6 query-specific SNVs were extracted to investigate the molecular epidemiological markers to trace back the outbreak between patients. The differences based on these 6 SNV sites can be visualized through a median-joining network using PopART software (Fig 4). The MIRU-VNTR loci of the four outbreak isolates (JP03, JP04, JP06 and JP07) were investigated prior to NGS, indicating that three additional isolates (JP01, JP02 and JP05) were closely related to the four outbreak isolates. The MIRU-VNTR results did not show sufficient discrimination among the seven isolates ranging from 2004 to 2011. NGS and TGS-TB analyses were performed to identify the SNVs among those isolates. No unique SNVs were detected for the four outbreak isolates (JP03, JP04, JP06 and JP07), and the two past isolates (JP01 and JP02) with two SNVs difference could be one outbreak source or potential ancestors related to this outbreak. It has been reported that the estimated rate of change in DNA sequences was 0.3–0.5 single nucleotide polymorphisms per genome per year [9, 13, 17, 18]; thus, the two detected SNVs represent a reasonable nucleotide substitution rate between the outbreak (2010/2011) and past isolates (2004/2005). In contrast, JP05 was isolated at a close time point, with four outbreak isolates in December 2010, and three SNV sites were detected when compared with four outbreak isolates. One additional IS*6110* insertion was detected in JP05 (Figs 4B and 5), strongly suggesting that JP05 did not appear to be involved in the outbreak. Such SNV networks facilitate the detection of epidemiological factors, and the obtained network is consistent with the field study (data not shown).

### In silico genotyping instead of conventional methods

In addition to Mtb genotyping using conventional methods, detection of the IS*6110* insertion (S3 Fig) and *in silico* spoligotyping (S4 Fig) are also available using the all-in-one TGS-TB analysis. *In silico* detection of the IS*6110* insertion provides precise information compared with IS*6110*-RFLP genotyping. The determination of the insertion sites and the orientation on the Mtb genome suggested the distinct type of genotypic evolution involving core genome SNVs, implying that novel Mtb clones can be easily detected as variations of the IS*6110* insertion. One of the Mtb isolates, JP05, showed a heterozygous population of the IS*6110* insertion sequencing, showing additional insertions at minor sites. Approximately 10% of the population might be mixed or contaminated in the original isolates, implying that the detection of the hetero-population could be valuable in distinguishing Mtb mixed-infections.

### Short MIRU-VNTR (sMIRU-VNTR)

MIRU-VNTR is a valuable discriminative genotyping tool, but NGS short reads are not a sufficient length to assign all loci of MIRU-VNTR (15 or 24 loci set). We originally assigned the 43 loci of short tandem repeats (TR), including 10 loci (MIRU02, ETRC, MIRU10, MIRU16, MIRU20, Mtub30, ETRB, MIRU27, Mtub34 and MIRU39) of the 24-loci MIRU-VNTR (S3 Table and S5 Fig). The selected loci are short TRs, designated as short MIRU-VNTRs (sMIRU-VNTRs). The detected repeats units are not always integers due to partial sequence repeat units.

## Conclusions

The results of *in silico* analysis through TGS-TB are consistent with conventional molecular genotyping methods (S6 Fig), suggesting that MiSeq NGS short reads could provide sufficient multiple genotypes at once to discriminate multiple strains of Mtb during an outbreak. The WGS approach is highly affordable for the characterization of the Mtb strains described above, but comprehensive genotyping tools, including traditional genotyping, have been expected to integrate all genetic information thus far. The all-in-one tool, TGS-TB, provides a more accurate and discriminative strain typing for clinical and epidemiological investigations. Thus, NGS strain typing could offer a total genotyping solution for Mtb outbreak and surveillance. The genotype information obtained for all Mtb isolates can be deposited into an integrated database for the future surveillance of outbreak and global infections.

## Materials and Methods

### Mycobacterium tuberculosis(Mtb) strains

The complete and draft genomic sequences for Mtb strains were retrieved from the GenBank database and NCBI SRA projects (ERP000111, ERP000192, ERP000276, ERP000436, ERP000520, ERP001567, ERP001731, ERP001885, ERP002611, SRP002589), and the following *in silico* analyses were performed using H37Rv (NC_000962.3) as a reference genome sequence. The performance and accuracy of TGS-TB were examined using seven Japanese Mtb isolates associated with local outbreaks (JP01, JP02, JP03, JP04, JP05, JP06 and JP07) (S1 Table).

### NGS

Genomic DNA from Mtb isolates was purified through the benzyl chloride method using the ISOPLANT kit (WAKO, Osaka, Japan). A genomic DNA library for NGS was prepared using the Nextera XT DNA Sample Prep Kit (Illumina, San Diego, CA, USA), followed by insert size selection using 1% TAE agarose electrophoresis to obtain an insert of approximately 600 bp. Sequencing was performed on MiSeq (Illumina, San Diego, CA, USA) using the MiSeq Reagent Kit v3 (600 cycle) with 350-mer x 250-mer paired-end short reads and 96-sample multiplexing (S1 Fig).

### KvarQ prediction

MTBC lineages/sublineages and potential AMRs were determined using the KvarQ script according to the manufacturer’s instructions [22]. The AMR target list has been improved to detect more reliable genetic alterations for the *embA*, *gyrA*, *katG*, *pncA*, *rpoB*, *rpsL*, *rrs* and *inhA* genes from TBDReaMDB [27] (S1 Text).

### Core-genome phylogenetic and linkage network analysis

Prior to *in silico* genotyping, the adapter sequences were trimmed from the short reads, and low quality bases with a Phred score less than 15 were eliminated using the Skewer program, to obtain sequences at least 50-mer in length [28]. The remaining reads are mapped using the BWA-mem program [29] with the Mtb H37Rv chromosome (NC_000962.3) reference genome sequence [30]. Reliable SNV sites with at least a 5x coverage depth and a Phred score of at least 20 were selected from the mapping. The SNV sites on the repeat regions of the H37RV genome (S2 Table), which was assigned based on the GenBank annotation file (NC_000962.3) and newly identified by the NUCmer program [31], was excluded for further core-genome phylogeny analysis because those SNVs sites are considered unreliable. A total of 21,805 SNV sites were extracted as an original dataset from Mtb with 12 complete genomes and 66 SRAs (S1 Table and Fig 2). Maximum likelihood phylogenetic analysis of all concatenated SNV alleles was performed using RAxML v8.2.0 [32] with 1,000 bootstrap iterations.

To investigate the epidemiological linkage between patients, the information from queries for isolate-specific genes or the above mentioned reference genomes can be downloaded as a nexus format file to visualize linkage networks, such as the median-joining network method using PopART (http://popart.otago.ac.nz).

### *In silico* detection of IS*6110* insertion site

The IS*6110* insertion site was detected in the following manner (Fig 5A): IS*6110* sequence positive short reads are collected from all short reads (Fig 5A1–2); the direction of collected reads was rearranged to the direction of the IS*6110* sequence (Fig 5A3); trimming and subtraction of the IS*6110* sequence (Acc.# X94955 and X94956) was performed according to an adapter trimming procedure (Fig 5A4–5); and the resulting trimmed short reads were mapped to the Mtb H37Rv chromosome (NC_000962.3) [30] through BWA-SW mapping [29] (Fig 5A6). The insertion site was detected at high coverage peaks on the chromosome DNA (Fig 5B).

### *In silico* spoligotyping

*In silico* spoligotyping was performed through a blastn search using 43 spacer sequences as a query [4] against the obtained short reads, and double mismatches with homology were considered a positive threshold.

### Short MIRU-VNTR

Instead of MIRU-VNTR [7, 19], we demonstrated that short VNTR loci can be used for further genotyping through short read sequencing. Possible candidates for additional VNTR loci with core sequences of ≥9 bp were extracted from the H37Rv genome according to the microorganisms tandem repeats database (http://minisatellites-rec.igmors.u-psud.fr/GPMS/) [33] using the default settings. Although NGS short reads are not sufficient for conventional MIRU-VNTR (MtbC15-9 type) because of the use of sequences up to 300 bp in length, 43 loci can be reasonably assigned as discriminative VNTR loci. Variable repeat units were assigned as follows: the corresponding short reads for respective VNTR loci were collected, followed by trimming of unique core genomic sequences at both ends of the TR. Using the remaining TR sequences, variable repeat units were counted based on the repeat core sequence. The selected loci were short repeat units, referred to as short MIRU-VNTR (sMIRU-VNTR) (S3 Table).

### Conventional molecular genotyping methods

Three molecular genotyping methods based on spoligotyping, VNTR and IS*6110*-RFLP were performed on the seven Japanese clinical strains (JP01 to JP07). Spoligotyping was performed according to the standardized protocol using an in-house membrane [4]. An optimized 24-loci MIRU-VNTR analysis [7] was conducted as previously described [26], and IS*6110*-RFLP was performed according to a standardized protocol [3].

## Supporting Information

S1 Fig Web image on the BaseSpace analysis for MiSeq sequencing. NGS was performed on a MiSeq NGS sequencer (Illumina, San Diego, CA, USA) using the MiSeq Reagent Kit v3 (600 cycle) with 350-mer x 250-mer paired-end short reads.
(PDF)

S2 Fig KvarQ prediction for lineages/sublineages and antimicrobial resistance. (PDF)

S3 Fig The results of the *in silico* detection of IS*6110* insertion sites. The red and sky-blue vertical bars on the H37Rv reference genome indicate the forward and reverse IS*6110* insertions, respectively.
(PDF)

S4 Fig The results of *in silico* spoligotyping using 43 spacer oligos. The filled circles indicate positive homology to each oligonucleotide sequence.
(PDF)

S5 Fig The results of *in silico* sMIRU-VNTR typing using 43 customized loci with tandem repeats (TRs). The detected number of TRs is shown on each locus and visualized using a color variation scale. Black and gray boxes indicate no detection of TRs and lower depths, respectively.
(PDF)

S6 Fig Conventional molecular genotyping results for the seven outbreak isolates examined in this study. (PDF)

S1 Table List of Mtb strains used in this study with information of the MTBC lineage/sublineage. (PDF)

S2 Table Repeat regions in the *M*. *tuberculosis* H37Rv genome (NC_000962.3). (PDF)

S3 Table Locus information for short MIRU-VNTR. (PDF)

S1 Text # Version: MTBC.niid.3.20150403 #. The AMR target list for KvarQ prediction has been improved to detect more reliable genetic alterations for the *embA*, *gyrA*, *katG*, *pncA*, *rpoB*, *rpsL*, *rrs* and *inhA* genes from TBDReaMDB [27].
(TXT)
