# Supplementary material for: TGS-TB: Total Genotyping Solution for Mycobacterium tuberculosis Using Short-Read Whole-Genome Sequencing
Source: PLoS One. 2015 Nov 13;10(11):e0142951. doi: 10.1371/journal.pone.0142951 (PMC4643978; doi:10.1371/journal.pone.0142951)
Supplement: S1 Fig — NGS was performed on a MiSeq NGS sequencer (Illumina, San Diego, CA, USA) using the MiSeq Reagent Kit v3 (600 cycle) with 350-mer x 250-mer paired-end short reads. (PDF) [file pone.0142951.s001.pdf]

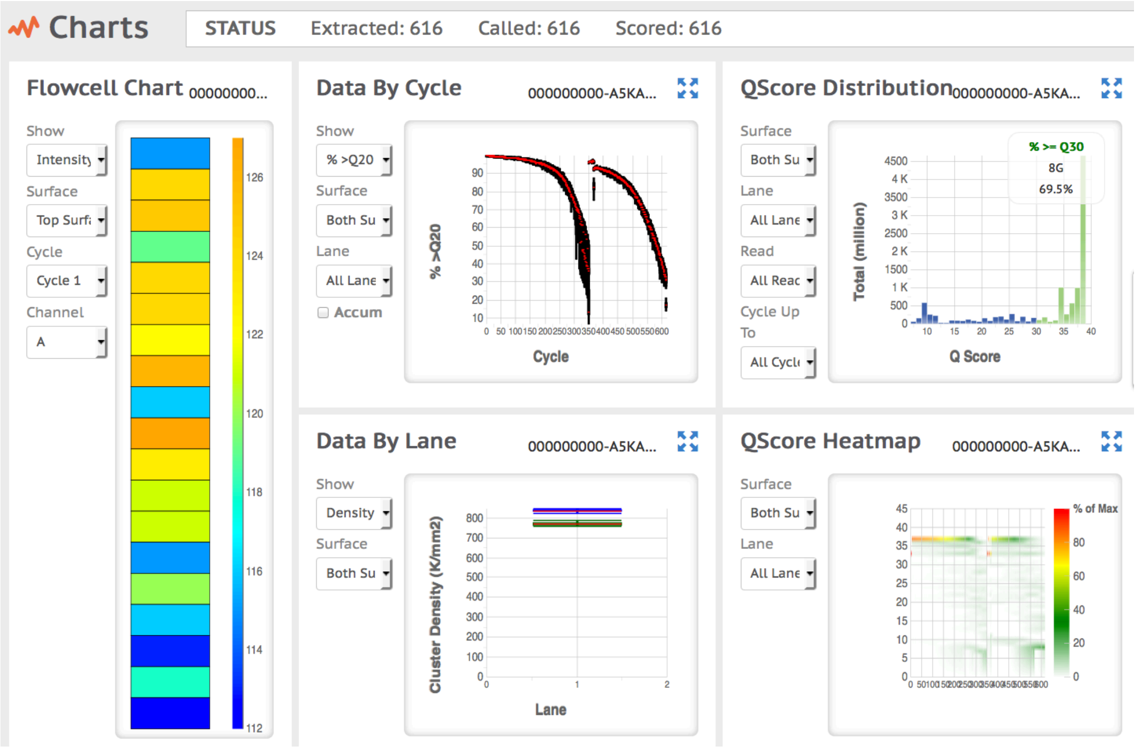

S1 Fig.

Front web image on the BaseSpace analysis for MiSeq sequencing. NGS was performed on a MiSeq NGS sequencer (Illumina, San Diego, CA, USA) using the MiSeq Reagent Kit v3 (600 cycle) with 350–mer x 250–mer paired-end short reads.
