# Supplementary material for: TGS-TB: Total Genotyping Solution for Mycobacterium tuberculosis Using Short-Read Whole-Genome Sequencing
Source: PLoS One. 2015 Nov 13;10(11):e0142951. doi: 10.1371/journal.pone.0142951 (PMC4643978; doi:10.1371/journal.pone.0142951)
Supplement: S2 Fig — (PDF) [file pone.0142951.s002.pdf]

KvarQ MTBC analysis result

Jump to KvarQ webpage

MTBC analysis version: MTBC.niid.3.20150403 about license

Sample	Phylo	Resistance
JP02	lineage 2/beijing sublineage	No resistance found
JP04	lineage 2/beijing sublineage	No resistance found
JP06	lineage 2/beijing sublineage	No resistance found
JP07	lineage 2/beijing sublineage	No resistance found
JP03	lineage 2/beijing sublineage	No resistance found
JP05	lineage 2/beijing sublineage	No resistance found
JP01	lineage 2/beijing sublineage	No resistance found

S2 Fig.

KvarQ prediction for lineages/sublineages and AMRs.
