# Supplementary material for: TGS-TB: Total Genotyping Solution for Mycobacterium tuberculosis Using Short-Read Whole-Genome Sequencing
Source: PLoS One. 2015 Nov 13;10(11):e0142951. doi: 10.1371/journal.pone.0142951 (PMC4643978; doi:10.1371/journal.pone.0142951)
Supplement: S3 Fig — The red and sky-blue vertical bars on the H37Rv reference genome indicate the forward and reverse IS6110 insertions, respectively. (PDF) [file pone.0142951.s003.pdf]

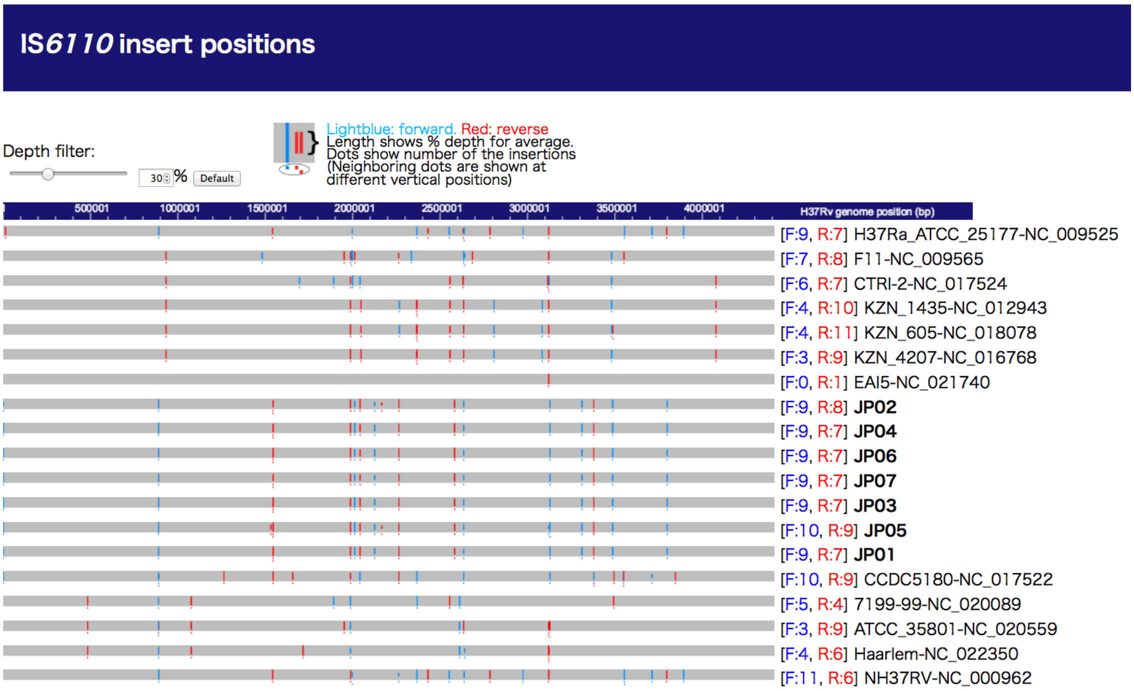

S3 Fig.

The results of the *in silico* detection of IS*6110* insertion sites. The red and sky-blue vertical bars on the H37Rv reference genome indicate the forward and reverse IS*6110* insertions, respectively.
