# Supplementary material for: TGS-TB: Total Genotyping Solution for Mycobacterium tuberculosis Using Short-Read Whole-Genome Sequencing
Source: PLoS One. 2015 Nov 13;10(11):e0142951. doi: 10.1371/journal.pone.0142951 (PMC4643978; doi:10.1371/journal.pone.0142951)
Supplement: S4 Fig — The filled circles indicate positive homology to each oligonucleotide sequence. (PDF) [file pone.0142951.s004.pdf]

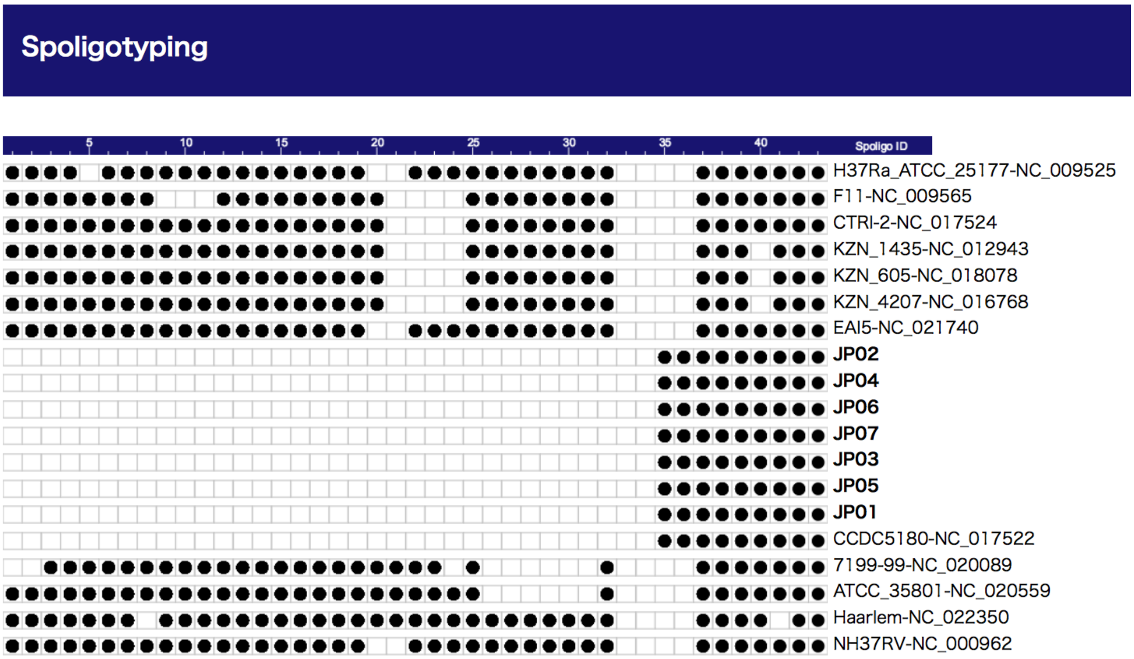

S4 Fig.

The results of *in silico* spoligotyping using 43 spacer oligos. The filled circles indicate positive homology to each oligo.
