# Supplementary material for: TGS-TB: Total Genotyping Solution for Mycobacterium tuberculosis Using Short-Read Whole-Genome Sequencing
Source: PLoS One. 2015 Nov 13;10(11):e0142951. doi: 10.1371/journal.pone.0142951 (PMC4643978; doi:10.1371/journal.pone.0142951)
Supplement: S5 Fig — The detected number of TRs is shown on each locus and visualized using a color variation scale. Black and gray boxes indicate no detection of TRs and lower depths, respectively. (PDF) [file pone.0142951.s005.pdf]

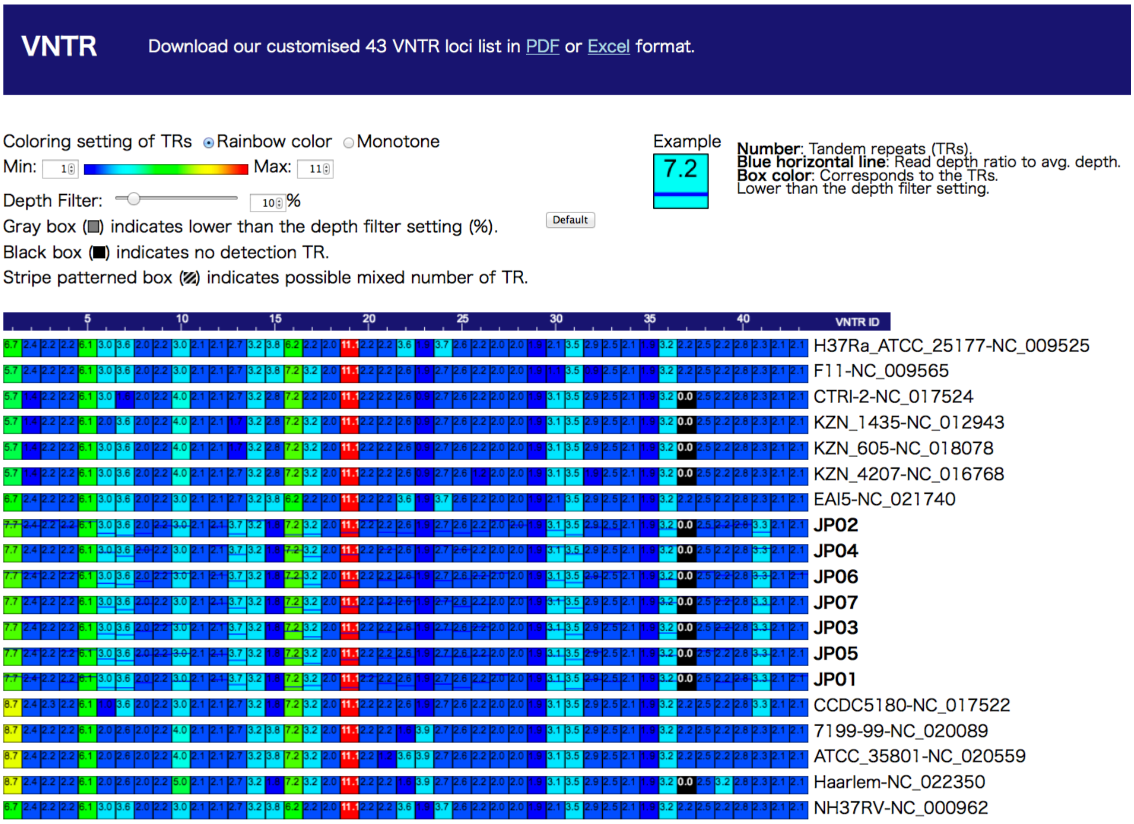

S5 Fig.

The results of *in silico* sMIRU-VNTR typing using 43 customized loci with tandem repeats (TRs). The detected number of TRs is shown on each locus and visualized using a colour variation scale. Black and grey boxes indicate no detection of TRs and lower depths, respectively.
