# Supplementary material for: TGS-TB: Total Genotyping Solution for Mycobacterium tuberculosis Using Short-Read Whole-Genome Sequencing
Source: PLoS One. 2015 Nov 13;10(11):e0142951. doi: 10.1371/journal.pone.0142951 (PMC4643978; doi:10.1371/journal.pone.0142951)

S6 Fig. Conventional molecular genotyping results for the seven outbreak isolates examined in this study.

Spoligotyping

Strain_ID	sp1	sp2	sp3	sp4	sp5	sp6	sp7	sp8	sp9	sp10	sp11	sp12	sp13	sp14	sp15	sp16	sp17	sp18	sp19	sp20	sp21	sp22	sp23	sp24	sp25	sp26	sp27	sp28	sp29	sp30	sp31	sp32	sp33	sp34	sp35	sp36	sp37	sp38	sp39	sp40	sp41	sp42	sp43
JP01	ND	ND	ND	ND	ND	ND	ND	ND	ND	ND	ND	ND	ND	ND	ND	ND	ND	ND	ND	ND	ND	ND	ND	ND	ND	ND	ND	ND	ND	ND	ND	ND	ND	ND	ND	ND	ND	ND	ND	ND	ND	ND	ND
JP02	0	0	0	0	0	0	0	0	0	0	0	0	0	0	0	0	0	0	0	0	0	0	0	0	0	0	0	0	0	0	0	0	0	0	1	1	1	1	1	1	1	1	1
JP03	0	0	0	0	0	0	0	0	0	0	0	0	0	0	0	0	0	0	0	0	0	0	0	0	0	0	0	0	0	0	0	0	0	0	1	1	1	1	1	1	1	1	1
JP04	0	0	0	0	0	0	0	0	0	0	0	0	0	0	0	0	0	0	0	0	0	0	0	0	0	0	0	0	0	0	0	0	0	0	1	1	1	1	1	1	1	1	1
JP05	0	0	0	0	0	0	0	0	0	0	0	0	0	0	0	0	0	0	0	0	0	0	0	0	0	0	0	0	0	0	0	0	0	0	1	1	1	1	1	1	1	1	1
JP06	0	0	0	0	0	0	0	0	0	0	0	0	0	0	0	0	0	0	0	0	0	0	0	0	0	0	0	0	0	0	0	0	0	0	1	1	1	1	1	1	1	1	1
JP07	0	0	0	0	0	0	0	0	0	0	0	0	0	0	0	0	0	0	0	0	0	0	0	0	0	0	0	0	0	0	0	0	0	0	1	1	1	1	1	1	1	1	1

VNTR

	0424	0960	1955	2074	2163b	2372	2996	3155	3192	3336	4052	4156	1982	2165	2163a	3820	4120	2347	2401	3171	3690	2461	0577	1644	0154	2059	2531	2687	3007	4348	0580	0802	1612	1895	3239
Strain_ID	Mtub04	MIRU10	Mtub21	Mtub24	QUB11b	VNTR 2372	MIRU26	QUB15	MIRU31	VNTR 3336	QUB26	VNTR 4156	VNTR 1982	ETR A	QUB11a	VNTR 3820	VNTR 4120	Mtub 29	Mtub 30	Mtub 34	Mtub 39	ETR-B	ETR-C	MIRU 16	MIRU 2	MIRU 20	MIRU 23	MIRU 24	MIRU 27	MIRU 39	MIRU 4	MIRU 40	QUB 23	VNTR 1895	ETR-F
JP01	2	3	3	3	3	3	7	4	2	4	8	4	9	4	7	17	15	4	2	3	3	2	4	3	2	2	5	1	3	3	2	3	7	4	3
JP02	2	3	3	3	3	3	7	4	2	4	8	4	9	4	7	17	15	4	2	3	3	2	4	3	2	2	5	1	3	3	2	3	7	4	3
JP03	2	3	3	3	3	3	7	4	2	4	8	4	9	4	7	17	15	4	2	3	3	2	4	3	2	2	5	1	3	3	2	3	7	4	3
JP04	2	3	3	3	3	3	7	4	2	4	8	4	9	4	7	17	15	4	2	3	3	2	4	3	2	2	5	1	3	3	2	3	7	4	3
JP05	2	3	3	3	3	3	7	4	2	4	8	4	9	4	7	17	15	4	2	3	3	2	4	3	2	2	5	1	3	3	2	3	7	4	3
JP06	2	3	3	3	3	3	7	4	2	4	8	4	9	4	7	17	16	4	2	3	3	2	4	3	2	2	5	1	3	3	2	3	7	4	3
JP07	2	3	3	3	3	3	7	4	2	4	8	4	9	4	7	17	15	4	2	3	3	2	4	3	2	2	5	1	3	3	2	3	7	4	3

IS*6110*-RFLP

Strain_ID
JP01
JP02
JP03
JP04
JP05
JP06
JP07

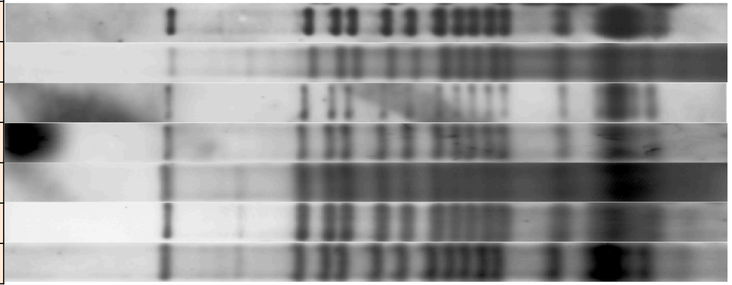

Supplement: S6 Fig — (PDF) [file pone.0142951.s006.pdf]
