# Supplementary material for: TGS-TB: Total Genotyping Solution for Mycobacterium tuberculosis Using Short-Read Whole-Genome Sequencing
Source: PLoS One. 2015 Nov 13;10(11):e0142951. doi: 10.1371/journal.pone.0142951 (PMC4643978; doi:10.1371/journal.pone.0142951)
Supplement: S1 Table — (PDF) [file pone.0142951.s007.pdf]

S1 Table. List of *M. tuberculosis* strains used in this study with information of the MTBC lineage/sublineage.

Strains ID	SRA_ID or GenBank_ID	Lineage
JP01	DRX037417	2.2.1
JP02	DRX037418	2.2.1
JP03	DRX037419	2.2.1
JP04	DRX037420	2.2.1
JP05	DRX037421	2.2.1
JP06	DRX037422	2.2.1
JP07	DRX037423	2.2.1
S00007279	ERR117694	1.1.1
S00019855	ERR387008	1.1.1.1
EAI5	NC_021740	1.1.2
S00007273	ERR114427	1.1.2
S00018368	ERR351915+ERR386847	1.1.3
S00013408	ERR278529+ERR386888	1.2.1
Bir_270	ERR046881	1.2.2
GX_450708	SRR1710066	2.1
GX_451017	SRR1710070	2.1 proto-Beijing
hlj100113	SRS475367	2.2.1
CCDC5180	NC_017522	2.2.1
08_0205	SRR1710073	2.2.1 MG2
SJ432	SRR1710110	2.2.1 MG3
0710Y	ERR117454	2.2.1 MG3
M08_14556	ERR015616	2.2.1 MG3
MTB_GT_333	ERR234208	2.2.1 MG3
-NA-	ERR019574	2.2.1 MG3
2533E	ERR234658	2.2.1 PG1
GQ366	ERR234133	2.2.1 PG1
L2_GQ1164	ERR234116	2.2.1 PG2
L2_GQ-1343	ERR234121	2.2.1.1
L2_N0130	ERR234263	2.2.1.2
10_0554	SRR1710083	2.2.2
Shanghai_09-1608	SRS790114	2.2.2 MG1
SJ649	SRR1710111	2.2.2 MG1
S00007361	ERR114513	3
S00007291	ERR114453	3.1.1
S00018322	ERR351869+ERR386801	3.1.2
S00007295	ERR117696	3.1.2.1
S00007298	ERR114442	3.1.2.2
H37Rv	NC_000962	4
S00017318	ERR330651	4
H37Ra_ATCC_25177	NC_009525	4

L4_DY195	ERR234201	4.1
Bir_74	ERR038277	4.1.1
S00007381	ERR114477	4.1.1.1
MT0016	SRR058116	4.1.1.2
S00007348	ERR117732	4.1.1.3
S00013476	ERR278597+ERR386956	4.1.2
7199-99	NC_020089	4.1.2.1
Haarlem	NC_022350	4.1.2.1
ATCC_35801	NC_020559	4.1.2.1
S00018390	ERR351937+ERR386869	4.1.2.1
S00009328	ERR228183	4.2.1
S00017375	ERR330708	4.2.2
S00009367	ERR228222	4.2.2.1
S00019824	ERR386977	4.3.1
S00013789	ERR294209	4.3.2
S00018339	ERR351886+ERR386818	4.3.2.1
F11	NC_009565	4.3.2.1
S00013400	ERR278521+ERR386880	4.3.3
CTRI-2	NC_017524	4.3.3
KZN_4207	NC_016768	4.3.3
KZN_605	NC_018078	4.3.3
KZN_1435	NC_012943	4.3.3
S00013459	ERR278580+ERR386939	4.3.4
S00013422	ERR278543+ERR386902	4.3.4.1
FFUL_KAUST_MTB26	ERR275206	4.3.4.2
S00013762	ERR294182	4.3.4.2.1
S00018378	ERR351925+ERR386857	4.4
S00013201	ERR270699	4.4.1
S00013203	ERR270701	4.4.1.1
Bir_79	ERR038282	4.4.1.2
S00013126	ERR270624	4.4.2
S00017336	ERR330669	4.5
S00018392	ERR351939+ERR386871	4.6
S00019843	ERR386996	4.6.1.1
S00007206	ERR123926	4.6.1.2
S00013416	ERR278537+ERR386896	4.6.2
Bir_225	ERR046839	4.6.2.1
S00007213	ERR123915	4.6.2.2
S00017343	ERR330676	4.7
S00013760	ERR294180	4.8
Bir_426	ERR072039	4.9
S00013829	ERR294249	5
L6_N0091	ERR234254	6
S00019848	ERR387001	BOV
Mt256	ERR181435	7
