# Supplementary material for: TGS-TB: Total Genotyping Solution for Mycobacterium tuberculosis Using Short-Read Whole-Genome Sequencing
Source: PLoS One. 2015 Nov 13;10(11):e0142951. doi: 10.1371/journal.pone.0142951 (PMC4643978; doi:10.1371/journal.pone.0142951)
Supplement: S2 Table — (PDF) [file pone.0142951.s008.pdf]

S2 Table. Repeat regions on M. tuberculosis H37Rv genome

Start		End	Note
23173	–	23273	H37Rv annotation (NC_000962.3)
79507	–	79551	H37Rv annotation (NC_000962.3)
80236	–	80550	H37Rv annotation (NC_000962.3)
103713	–	105215	H37Rv annotation (NC_000962.3)
154073	–	154125	H37Rv annotation (NC_000962.3)
154126	–	154178	H37Rv annotation (NC_000962.3)
154179	–	154231	H37Rv annotation (NC_000962.3)
206812	–	206850	H37Rv annotation (NC_000962.3)
206869	–	206907	H37Rv annotation (NC_000962.3)
272855	–	272955	H37Rv annotation (NC_000962.3)
547488	–	547517	H37Rv annotation (NC_000962.3)
580578	–	580654	H37Rv annotation (NC_000962.3)
580655	–	580731	H37Rv annotation (NC_000962.3)
580732	–	580808	H37Rv annotation (NC_000962.3)
616828	–	616878	H37Rv annotation (NC_000962.3)
642754	–	642811	H37Rv annotation (NC_000962.3)
701247	–	701369	H37Rv annotation (NC_000962.3)
703912	–	703985	H37Rv annotation (NC_000962.3)
706790	–	706863	H37Rv annotation (NC_000962.3)
709425	–	709548	H37Rv annotation (NC_000962.3)
709585	–	709663	H37Rv annotation (NC_000962.3)
711624	–	711702	H37Rv annotation (NC_000962.3)
795467	–	795518	H37Rv annotation (NC_000962.3)
802429	–	802477	H37Rv annotation (NC_000962.3)
812835	–	812921	H37Rv annotation (NC_000962.3)
812922	–	812975	H37Rv annotation (NC_000962.3)
863155	–	863255	H37Rv annotation (NC_000962.3)
889017	–	889020	H37Rv annotation (NC_000962.3)
889021	–	889048	H37Rv annotation (NC_000962.3)
890348	–	890375	H37Rv annotation (NC_000962.3)
890376	–	890379	H37Rv annotation (NC_000962.3)
960173	–	960225	H37Rv annotation (NC_000962.3)
960226	–	960278	H37Rv annotation (NC_000962.3)
960279	–	960333	H37Rv annotation (NC_000962.3)
1025458	–	1025472	H37Rv annotation (NC_000962.3)
1026879	–	1026893	H37Rv annotation (NC_000962.3)
1027061	–	1027076	H37Rv annotation (NC_000962.3)
1029345	–	1029360	H37Rv annotation (NC_000962.3)
1164572	–	1164589	H37Rv annotation (NC_000962.3)
1165532	–	1165549	H37Rv annotation (NC_000962.3)
1179345	–	1179395	H37Rv annotation (NC_000962.3)
1251621	–	1252945	H37Rv annotation (NC_000962.3)
1276296	–	1277643	H37Rv annotation (NC_000962.3)
1277843	–	1277846	H37Rv annotation (NC_000962.3)
1277847	–	1277863	H37Rv annotation (NC_000962.3)
1278800	–	1278816	H37Rv annotation (NC_000962.3)
1278817	–	1278820	H37Rv annotation (NC_000962.3)
1305495	–	1305556	H37Rv annotation (NC_000962.3)
1305557	–	1305618	H37Rv annotation (NC_000962.3)
1305619	–	1305661	H37Rv annotation (NC_000962.3)
1456585	–	1456627	H37Rv annotation (NC_000962.3)
1457453	–	1457504	H37Rv annotation (NC_000962.3)
1457505	–	1457557	H37Rv annotation (NC_000962.3)
1468143	–	1468161	H37Rv annotation (NC_000962.3)
1469633	–	1469651	H37Rv annotation (NC_000962.3)
1507531	–	1507581	H37Rv annotation (NC_000962.3)
1541949	–	1541951	H37Rv annotation (NC_000962.3)

1543307	–	1543309	H37Rv annotation (NC_000962.3)
1612558	–	1612578	H37Rv annotation (NC_000962.3)
1612579	–	1612599	H37Rv annotation (NC_000962.3)
1612600	–	1612620	H37Rv annotation (NC_000962.3)
1612621	–	1612641	H37Rv annotation (NC_000962.3)
1612642	–	1612662	H37Rv annotation (NC_000962.3)
1625366	–	1625418	H37Rv annotation (NC_000962.3)
1633531	–	1634790	H37Rv annotation (NC_000962.3)
1637133	–	1638392	H37Rv annotation (NC_000962.3)
1644261	–	1644313	H37Rv annotation (NC_000962.3)
1644314	–	1644364	H37Rv annotation (NC_000962.3)
1779266	–	1779277	H37Rv annotation (NC_000962.3)
1779959	–	1780047	H37Rv annotation (NC_000962.3)
1780485	–	1780573	H37Rv annotation (NC_000962.3)
1788514	–	1789811	H37Rv annotation (NC_000962.3)
1788514	–	1788525	H37Rv annotation (NC_000962.3)
1830074	–	1830125	H37Rv annotation (NC_000962.3)
1907460	–	1907515	H37Rv annotation (NC_000962.3)
1907516	–	1907571	H37Rv annotation (NC_000962.3)
1927218	–	1928589	H37Rv annotation (NC_000962.3)
1938093	–	1938145	H37Rv annotation (NC_000962.3)
1944756	–	1944808	H37Rv annotation (NC_000962.3)
1982887	–	1982964	H37Rv annotation (NC_000962.3)
1982965	–	1983042	H37Rv annotation (NC_000962.3)
1983043	–	1983120	H37Rv annotation (NC_000962.3)
1983121	–	1983198	H37Rv annotation (NC_000962.3)
1983199	–	1983276	H37Rv annotation (NC_000962.3)
1983277	–	1983354	H37Rv annotation (NC_000962.3)
1987703	–	1987730	H37Rv annotation (NC_000962.3)
1989030	–	1989057	H37Rv annotation (NC_000962.3)
1996101	–	1996128	H37Rv annotation (NC_000962.3)
1997428	–	1997455	H37Rv annotation (NC_000962.3)
1998584	–	1998597	H37Rv annotation (NC_000962.3)
1999800	–	1999813	H37Rv annotation (NC_000962.3)
2059441	–	2059498	H37Rv annotation (NC_000962.3)
2059518	–	2059575	H37Rv annotation (NC_000962.3)
2163323	–	2163392	H37Rv annotation (NC_000962.3)
2163393	–	2163461	H37Rv annotation (NC_000962.3)
2163462	–	2163530	H37Rv annotation (NC_000962.3)
2163741	–	2163809	H37Rv annotation (NC_000962.3)
2163810	–	2163878	H37Rv annotation (NC_000962.3)
2163879	–	2163947	H37Rv annotation (NC_000962.3)
2163948	–	2164016	H37Rv annotation (NC_000962.3)
2164017	–	2164085	H37Rv annotation (NC_000962.3)
2195989	–	2197350	H37Rv annotation (NC_000962.3)
2330147	–	2330225	H37Rv annotation (NC_000962.3)
2365414	–	2365441	H37Rv annotation (NC_000962.3)
2366741	–	2366768	H37Rv annotation (NC_000962.3)
2372437	–	2372492	H37Rv annotation (NC_000962.3)
2372494	–	2372549	H37Rv annotation (NC_000962.3)
2430117	–	2430144	H37Rv annotation (NC_000962.3)
2431444	–	2431471	H37Rv annotation (NC_000962.3)
2458392	–	2458449	H37Rv annotation (NC_000962.3)
2493801	–	2493818	H37Rv annotation (NC_000962.3)
2522173	–	2522230	H37Rv annotation (NC_000962.3)
2523184	–	2523236	H37Rv annotation (NC_000962.3)
2531898	–	2531950	H37Rv annotation (NC_000962.3)
2531951	–	2532003	H37Rv annotation (NC_000962.3)

2532004	–	2532056	H37Rv annotation (NC_000962.3)
2532057	–	2532109	H37Rv annotation (NC_000962.3)
2532110	–	2532162	H37Rv annotation (NC_000962.3)
2532163	–	2532212	H37Rv annotation (NC_000962.3)
2550011	–	2550013	H37Rv annotation (NC_000962.3)
2550014	–	2550041	H37Rv annotation (NC_000962.3)
2551341	–	2551368	H37Rv annotation (NC_000962.3)
2551369	–	2551371	H37Rv annotation (NC_000962.3)
2635577	–	2635604	H37Rv annotation (NC_000962.3)
2636904	–	2636931	H37Rv annotation (NC_000962.3)
2687128	–	2687179	H37Rv annotation (NC_000962.3)
2687180	–	2687257	H37Rv annotation (NC_000962.3)
2716315	–	2716391	H37Rv annotation (NC_000962.3)
2720644	–	2720656	H37Rv annotation (NC_000962.3)
2721844	–	2721856	H37Rv annotation (NC_000962.3)
2762762	–	2763061	H37Rv annotation (NC_000962.3)
2763397	–	2763696	H37Rv annotation (NC_000962.3)
2784614	–	2784642	H37Rv annotation (NC_000962.3)
2785942	–	2785970	H37Rv annotation (NC_000962.3)
2800671	–	2800918	H37Rv annotation (NC_000962.3)
2806368	–	2806625	H37Rv annotation (NC_000962.3)
2972106	–	2972108	H37Rv annotation (NC_000962.3)
2972109	–	2972136	H37Rv annotation (NC_000962.3)
2973436	–	2973463	H37Rv annotation (NC_000962.3)
2973464	–	2973466	H37Rv annotation (NC_000962.3)
2983019	–	2983033	H37Rv annotation (NC_000962.3)
2996003	–	2996053	H37Rv annotation (NC_000962.3)
2996054	–	2996104	H37Rv annotation (NC_000962.3)
2996105	–	2996155	H37Rv annotation (NC_000962.3)
3007063	–	3007115	H37Rv annotation (NC_000962.3)
3007116	–	3007168	H37Rv annotation (NC_000962.3)
3007169	–	3007221	H37Rv annotation (NC_000962.3)
3013612	–	3013687	H37Rv annotation (NC_000962.3)
3073055	–	3073112	H37Rv annotation (NC_000962.3)
3119185	–	3123576	H37Rv annotation (NC_000962.3)
3119185	–	3119220	H37Rv annotation (NC_000962.3)
3119259	–	3119294	H37Rv annotation (NC_000962.3)
3119335	–	3119370	H37Rv annotation (NC_000962.3)
3119411	–	3119446	H37Rv annotation (NC_000962.3)
3119484	–	3119519	H37Rv annotation (NC_000962.3)
3119556	–	3119591	H37Rv annotation (NC_000962.3)
3119627	–	3119662	H37Rv annotation (NC_000962.3)
3119701	–	3119736	H37Rv annotation (NC_000962.3)
3119777	–	3119812	H37Rv annotation (NC_000962.3)
3119848	–	3119883	H37Rv annotation (NC_000962.3)
3119921	–	3119956	H37Rv annotation (NC_000962.3)
3119995	–	3120030	H37Rv annotation (NC_000962.3)
3120068	–	3120103	H37Rv annotation (NC_000962.3)
3120141	–	3120176	H37Rv annotation (NC_000962.3)
3120213	–	3120248	H37Rv annotation (NC_000962.3)
3120285	–	3120320	H37Rv annotation (NC_000962.3)
3120359	–	3120394	H37Rv annotation (NC_000962.3)
3120433	–	3120468	H37Rv annotation (NC_000962.3)
3120504	–	3120523	H37Rv annotation (NC_000962.3)
3121882	–	3121897	H37Rv annotation (NC_000962.3)
3121938	–	3121973	H37Rv annotation (NC_000962.3)
3122013	–	3122048	H37Rv annotation (NC_000962.3)
3122086	–	3122121	H37Rv annotation (NC_000962.3)

3122158	–	3122193	H37Rv annotation (NC_000962.3)
3122230	–	3122265	H37Rv annotation (NC_000962.3)
3122303	–	3122338	H37Rv annotation (NC_000962.3)
3122375	–	3122410	H37Rv annotation (NC_000962.3)
3122436	–	3122471	H37Rv annotation (NC_000962.3)
3122513	–	3122548	H37Rv annotation (NC_000962.3)
3122585	–	3122620	H37Rv annotation (NC_000962.3)
3122661	–	3122696	H37Rv annotation (NC_000962.3)
3122738	–	3122773	H37Rv annotation (NC_000962.3)
3122811	–	3122846	H37Rv annotation (NC_000962.3)
3122882	–	3122917	H37Rv annotation (NC_000962.3)
3122955	–	3122990	H37Rv annotation (NC_000962.3)
3123029	–	3123064	H37Rv annotation (NC_000962.3)
3123102	–	3123137	H37Rv annotation (NC_000962.3)
3123173	–	3123208	H37Rv annotation (NC_000962.3)
3123248	–	3123283	H37Rv annotation (NC_000962.3)
3123318	–	3123353	H37Rv annotation (NC_000962.3)
3123390	–	3123425	H37Rv annotation (NC_000962.3)
3123467	–	3123502	H37Rv annotation (NC_000962.3)
3123541	–	3123576	H37Rv annotation (NC_000962.3)
3155874	–	3155927	H37Rv annotation (NC_000962.3)
3155928	–	3155981	H37Rv annotation (NC_000962.3)
3155982	–	3156035	H37Rv annotation (NC_000962.3)
3156036	–	3156089	H37Rv annotation (NC_000962.3)
3160522	–	3160583	H37Rv annotation (NC_000962.3)
3171468	–	3171518	H37Rv annotation (NC_000962.3)
3171522	–	3171572	H37Rv annotation (NC_000962.3)
3171576	–	3171616	H37Rv annotation (NC_000962.3)
3181794	–	3181836	H37Rv annotation (NC_000962.3)
3192202	–	3192254	H37Rv annotation (NC_000962.3)
3192255	–	3192307	H37Rv annotation (NC_000962.3)
3192308	–	3192360	H37Rv annotation (NC_000962.3)
3318835	–	3318889	H37Rv annotation (NC_000962.3)
3319468	–	3319568	H37Rv annotation (NC_000962.3)
3319569	–	3319666	H37Rv annotation (NC_000962.3)
3333768	–	3333773	H37Rv annotation (NC_000962.3)
3335787	–	3335792	H37Rv annotation (NC_000962.3)
3381351	–	3381365	H37Rv annotation (NC_000962.3)
3382660	–	3382674	H37Rv annotation (NC_000962.3)
3481399	–	3481413	H37Rv annotation (NC_000962.3)
3482708	–	3482722	H37Rv annotation (NC_000962.3)
3551227	–	3551229	H37Rv annotation (NC_000962.3)
3551230	–	3551257	H37Rv annotation (NC_000962.3)
3552557	–	3552584	H37Rv annotation (NC_000962.3)
3552585	–	3552587	H37Rv annotation (NC_000962.3)
3552710	–	3552712	H37Rv annotation (NC_000962.3)
3552713	–	3552740	H37Rv annotation (NC_000962.3)
3554040	–	3554067	H37Rv annotation (NC_000962.3)
3554068	–	3554070	H37Rv annotation (NC_000962.3)
3591493	–	3591569	H37Rv annotation (NC_000962.3)
3626614	–	3626666	H37Rv annotation (NC_000962.3)
3658658	–	3658715	H37Rv annotation (NC_000962.3)
3704895	–	3705004	H37Rv annotation (NC_000962.3)
3710382	–	3710409	H37Rv annotation (NC_000962.3)
3711709	–	3711736	H37Rv annotation (NC_000962.3)
3743198	–	3743404	H37Rv annotation (NC_000962.3)
3743402	–	3743510	H37Rv annotation (NC_000962.3)
3743508	–	3743605	H37Rv annotation (NC_000962.3)

3769514	–	3769720	H37Rv annotation (NC_000962.3)
3769754	–	3769862	H37Rv annotation (NC_000962.3)
3770994	–	3771091	H37Rv annotation (NC_000962.3)
3795058	–	3795085	H37Rv annotation (NC_000962.3)
3796385	–	3796412	H37Rv annotation (NC_000962.3)
3799987	–	3800011	H37Rv annotation (NC_000962.3)
3801530	–	3801554	H37Rv annotation (NC_000962.3)
3883550	–	3884921	H37Rv annotation (NC_000962.3)
3890779	–	3890806	H37Rv annotation (NC_000962.3)
3892106	–	3892133	H37Rv annotation (NC_000962.3)
3945098	–	3945597	H37Rv annotation (NC_000962.3)
3950830	–	3951329	H37Rv annotation (NC_000962.3)
3991568	–	3991625	H37Rv annotation (NC_000962.3)
4052949	–	4052966	H37Rv annotation (NC_000962.3)
4052971	–	4052994	H37Rv annotation (NC_000962.3)
4052995	–	4053105	H37Rv annotation (NC_000962.3)
4053004	–	4053021	H37Rv annotation (NC_000962.3)
4053106	–	4053216	H37Rv annotation (NC_000962.3)
4053217	–	4053327	H37Rv annotation (NC_000962.3)
4053328	–	4053438	H37Rv annotation (NC_000962.3)
4053439	–	4053549	H37Rv annotation (NC_000962.3)
4075615	–	4075630	H37Rv annotation (NC_000962.3)
4077735	–	4077750	H37Rv annotation (NC_000962.3)
4078506	–	4078518	H37Rv annotation (NC_000962.3)
4079786	–	4079798	H37Rv annotation (NC_000962.3)
4134601	–	4134725	H37Rv annotation (NC_000962.3)
4348721	–	4348773	H37Rv annotation (NC_000962.3)
4348774	–	4348826	H37Rv annotation (NC_000962.3)
4353280	–	4353330	H37Rv annotation (NC_000962.3)
4353331	–	4353381	H37Rv annotation (NC_000962.3)
4353382	–	4353432	H37Rv annotation (NC_000962.3)
80185	–	80373	NUCmer repeat finding (this study)
80927	–	82664	NUCmer repeat finding (this study)
103706	–	105076	NUCmer repeat finding (this study)
103780	–	105130	NUCmer repeat finding (this study)
149572	–	149762	NUCmer repeat finding (this study)
154115	–	154186	NUCmer repeat finding (this study)
154168	–	154239	NUCmer repeat finding (this study)
177055	–	177175	NUCmer repeat finding (this study)
177169	–	177451	NUCmer repeat finding (this study)
197824	–	198205	NUCmer repeat finding (this study)
309030	–	309532	NUCmer repeat finding (this study)
309899	–	310101	NUCmer repeat finding (this study)
332699	–	333610	NUCmer repeat finding (this study)
333511	–	333734	NUCmer repeat finding (this study)
335829	–	336275	NUCmer repeat finding (this study)
335417	–	336379	NUCmer repeat finding (this study)
336566	–	336788	NUCmer repeat finding (this study)
338214	–	338450	NUCmer repeat finding (this study)
338298	–	338537	NUCmer repeat finding (this study)
338201	–	339142	NUCmer repeat finding (this study)
339373	–	339875	NUCmer repeat finding (this study)
366867	–	367146	NUCmer repeat finding (this study)
368076	–	368602	NUCmer repeat finding (this study)
369390	–	369805	NUCmer repeat finding (this study)
370548	–	370963	NUCmer repeat finding (this study)
371821	–	372375	NUCmer repeat finding (this study)
372246	–	372525	NUCmer repeat finding (this study)

374567	–	374856	NUCmer repeat finding (this study)
374504	–	375058	NUCmer repeat finding (this study)
374593	–	375119	NUCmer repeat finding (this study)
400152	–	401701	NUCmer repeat finding (this study)
428385	–	428757	NUCmer repeat finding (this study)
428368	–	428760	NUCmer repeat finding (this study)
429620	–	429909	NUCmer repeat finding (this study)
424012	–	429920	NUCmer repeat finding (this study)
429766	–	430167	NUCmer repeat finding (this study)
429648	–	430190	NUCmer repeat finding (this study)
429662	–	430194	NUCmer repeat finding (this study)
431159	–	431541	NUCmer repeat finding (this study)
431025	–	431567	NUCmer repeat finding (this study)
432541	–	432929	NUCmer repeat finding (this study)
432401	–	432933	NUCmer repeat finding (this study)
460808	–	461483	NUCmer repeat finding (this study)
473744	–	474099	NUCmer repeat finding (this study)
475810	–	476177	NUCmer repeat finding (this study)
562307	–	562394	NUCmer repeat finding (this study)
569821	–	569922	NUCmer repeat finding (this study)
569877	–	569978	NUCmer repeat finding (this study)
577286	–	577438	NUCmer repeat finding (this study)
577344	–	577496	NUCmer repeat finding (this study)
580577	–	580737	NUCmer repeat finding (this study)
580654	–	580814	NUCmer repeat finding (this study)
606511	–	608060	NUCmer repeat finding (this study)
675486	–	675612	NUCmer repeat finding (this study)
675492	–	675653	NUCmer repeat finding (this study)
686110	–	686491	NUCmer repeat finding (this study)
798824	–	799109	NUCmer repeat finding (this study)
832045	–	832356	NUCmer repeat finding (this study)
838771	–	838896	NUCmer repeat finding (this study)
838486	–	838926	NUCmer repeat finding (this study)
840739	–	841665	NUCmer repeat finding (this study)
842020	–	842331	NUCmer repeat finding (this study)
889020	–	890375	NUCmer repeat finding (this study)
889021	–	891461	NUCmer repeat finding (this study)
908181	–	909346	NUCmer repeat finding (this study)
917611	–	917677	NUCmer repeat finding (this study)
917669	–	917735	NUCmer repeat finding (this study)
921744	–	921892	NUCmer repeat finding (this study)
921580	–	921893	NUCmer repeat finding (this study)
925280	–	925404	NUCmer repeat finding (this study)
960167	–	960270	NUCmer repeat finding (this study)
960213	–	960323	NUCmer repeat finding (this study)
960228	–	960324	NUCmer repeat finding (this study)
960175	–	960325	NUCmer repeat finding (this study)
960220	–	960327	NUCmer repeat finding (this study)
964605	–	964965	NUCmer repeat finding (this study)
964770	–	965086	NUCmer repeat finding (this study)
1093336	–	1093666	NUCmer repeat finding (this study)
1093422	–	1093866	NUCmer repeat finding (this study)
1093841	–	1094592	NUCmer repeat finding (this study)
1095120	–	1095317	NUCmer repeat finding (this study)
1095175	–	1095393	NUCmer repeat finding (this study)
1095088	–	1095413	NUCmer repeat finding (this study)
1095372	–	1095566	NUCmer repeat finding (this study)
1095301	–	1095739	NUCmer repeat finding (this study)

1095936	–	1096687	NUCmer repeat finding (this study)
1095821	–	1096692	NUCmer repeat finding (this study)
1160544	–	1161151	NUCmer repeat finding (this study)
1160539	–	1161165	NUCmer repeat finding (this study)
1160546	–	1161167	NUCmer repeat finding (this study)
1164572	–	1165549	NUCmer repeat finding (this study)
1169297	–	1170206	NUCmer repeat finding (this study)
1169298	–	1170733	NUCmer repeat finding (this study)
1189340	–	1189539	NUCmer repeat finding (this study)
1190000	–	1190120	NUCmer repeat finding (this study)
1189730	–	1190175	NUCmer repeat finding (this study)
1191259	–	1191467	NUCmer repeat finding (this study)
1191724	–	1191882	NUCmer repeat finding (this study)
1191454	–	1191899	NUCmer repeat finding (this study)
1211532	–	1212158	NUCmer repeat finding (this study)
1216792	–	1217061	NUCmer repeat finding (this study)
1216441	–	1217067	NUCmer repeat finding (this study)
1275925	–	1276053	NUCmer repeat finding (this study)
1276293	–	1277797	NUCmer repeat finding (this study)
1277843	–	1278820	NUCmer repeat finding (this study)
1287355	–	1288094	NUCmer repeat finding (this study)
1305495	–	1305599	NUCmer repeat finding (this study)
1305557	–	1305661	NUCmer repeat finding (this study)
1339350	–	1340433	NUCmer repeat finding (this study)
1340642	–	1341288	NUCmer repeat finding (this study)
1340495	–	1341292	NUCmer repeat finding (this study)
1341296	–	1342730	NUCmer repeat finding (this study)
1357423	–	1357613	NUCmer repeat finding (this study)
1441366	–	1441638	NUCmer repeat finding (this study)
1441519	–	1441791	NUCmer repeat finding (this study)
1451779	–	1451994	NUCmer repeat finding (this study)
1451836	–	1451995	NUCmer repeat finding (this study)
1451778	–	1451998	NUCmer repeat finding (this study)
1468166	–	1469616	NUCmer repeat finding (this study)
1479356	–	1480072	NUCmer repeat finding (this study)
1481030	–	1481746	NUCmer repeat finding (this study)
1532540	–	1533653	NUCmer repeat finding (this study)
1541949	–	1543307	NUCmer repeat finding (this study)
1541952	–	1543318	NUCmer repeat finding (this study)
1633311	–	1633538	NUCmer repeat finding (this study)
1633531	–	1634790	NUCmer repeat finding (this study)
1636706	–	1636933	NUCmer repeat finding (this study)
1637133	–	1638392	NUCmer repeat finding (this study)
1644263	–	1644365	NUCmer repeat finding (this study)
1702677	–	1702843	NUCmer repeat finding (this study)
1702707	–	1702849	NUCmer repeat finding (this study)
1773962	–	1774031	NUCmer repeat finding (this study)
1774037	–	1774106	NUCmer repeat finding (this study)
1779809	–	1780054	NUCmer repeat finding (this study)
1779984	–	1780311	NUCmer repeat finding (this study)
1780241	–	1780580	NUCmer repeat finding (this study)
1780641	–	1782817	NUCmer repeat finding (this study)
1785877	–	1786318	NUCmer repeat finding (this study)
1788514	–	1789865	NUCmer repeat finding (this study)
1814895	–	1815098	NUCmer repeat finding (this study)
1814853	–	1815128	NUCmer repeat finding (this study)
1876112	–	1876668	NUCmer repeat finding (this study)
1875622	–	1876899	NUCmer repeat finding (this study)

1879291	–	1879378	NUCmer repeat finding (this study)
1882019	–	1883293	NUCmer repeat finding (this study)
1895353	–	1895534	NUCmer repeat finding (this study)
1895410	–	1895591	NUCmer repeat finding (this study)
1946849	–	1947023	NUCmer repeat finding (this study)
1963420	–	1964160	NUCmer repeat finding (this study)
1982887	–	1983273	NUCmer repeat finding (this study)
1983043	–	1983350	NUCmer repeat finding (this study)
1982013	–	1983351	NUCmer repeat finding (this study)
1987699	–	1989057	NUCmer repeat finding (this study)
1987702	–	1989058	NUCmer repeat finding (this study)
1987703	–	1989059	NUCmer repeat finding (this study)
1996101	–	1997456	NUCmer repeat finding (this study)
1996100	–	1997458	NUCmer repeat finding (this study)
1996099	–	1997463	NUCmer repeat finding (this study)
1997453	–	1998612	NUCmer repeat finding (this study)
2000927	–	2001050	NUCmer repeat finding (this study)
2000930	–	2001056	NUCmer repeat finding (this study)
2025302	–	2026163	NUCmer repeat finding (this study)
2028426	–	2029242	NUCmer repeat finding (this study)
2030347	–	2030978	NUCmer repeat finding (this study)
2030332	–	2030983	NUCmer repeat finding (this study)
2059430	–	2059523	NUCmer repeat finding (this study)
2059507	–	2059600	NUCmer repeat finding (this study)
2062007	–	2062263	NUCmer repeat finding (this study)
2062400	–	2062639	NUCmer repeat finding (this study)
2074436	–	2074577	NUCmer repeat finding (this study)
2074492	–	2074633	NUCmer repeat finding (this study)
2089244	–	2089483	NUCmer repeat finding (this study)
2134912	–	2135764	NUCmer repeat finding (this study)
2163328	–	2163461	NUCmer repeat finding (this study)
2163397	–	2163530	NUCmer repeat finding (this study)
2163731	–	2164023	NUCmer repeat finding (this study)
2163800	–	2164092	NUCmer repeat finding (this study)
2165327	–	2165508	NUCmer repeat finding (this study)
2165402	–	2165583	NUCmer repeat finding (this study)
2195857	–	2197360	NUCmer repeat finding (this study)
2198687	–	2198815	NUCmer repeat finding (this study)
2262010	–	2263169	NUCmer repeat finding (this study)
2294817	–	2296422	NUCmer repeat finding (this study)
2299556	–	2300118	NUCmer repeat finding (this study)
2299197	–	2300822	NUCmer repeat finding (this study)
2300913	–	2302512	NUCmer repeat finding (this study)
2305625	–	2306187	NUCmer repeat finding (this study)
2305266	–	2306889	NUCmer repeat finding (this study)
2337937	–	2338094	NUCmer repeat finding (this study)
2340530	–	2340687	NUCmer repeat finding (this study)
2347380	–	2347554	NUCmer repeat finding (this study)
2347391	–	2347611	NUCmer repeat finding (this study)
2347452	–	2347613	NUCmer repeat finding (this study)
2358503	–	2359351	NUCmer repeat finding (this study)
2359727	–	2359994	NUCmer repeat finding (this study)
2365404	–	2366768	NUCmer repeat finding (this study)
2365414	–	2366769	NUCmer repeat finding (this study)
2365411	–	2366771	NUCmer repeat finding (this study)
2430115	–	2431471	NUCmer repeat finding (this study)
2430116	–	2431472	NUCmer repeat finding (this study)
2430117	–	2431473	NUCmer repeat finding (this study)

2430114	–	2431474	NUCmer repeat finding (this study)
2439140	–	2439948	NUCmer repeat finding (this study)
2461320	–	2461447	NUCmer repeat finding (this study)
2461378	–	2461504	NUCmer repeat finding (this study)
2531900	–	2532002	NUCmer repeat finding (this study)
2531893	–	2532156	NUCmer repeat finding (this study)
2531946	–	2532209	NUCmer repeat finding (this study)
2550012	–	2551368	NUCmer repeat finding (this study)
2550006	–	2551369	NUCmer repeat finding (this study)
2550010	–	2551370	NUCmer repeat finding (this study)
2625890	–	2626536	NUCmer repeat finding (this study)
2625886	–	2626683	NUCmer repeat finding (this study)
2635145	–	2635576	NUCmer repeat finding (this study)
2635575	–	2636931	NUCmer repeat finding (this study)
2635577	–	2636932	NUCmer repeat finding (this study)
2635576	–	2636937	NUCmer repeat finding (this study)
2636930	–	2637028	NUCmer repeat finding (this study)
2637300	–	2637501	NUCmer repeat finding (this study)
2637258	–	2637533	NUCmer repeat finding (this study)
2637164	–	2637535	NUCmer repeat finding (this study)
2638293	–	2638724	NUCmer repeat finding (this study)
2639035	–	2639531	NUCmer repeat finding (this study)
2639515	–	2639613	NUCmer repeat finding (this study)
2720635	–	2721442	NUCmer repeat finding (this study)
2784601	–	2785969	NUCmer repeat finding (this study)
2784615	–	2785970	NUCmer repeat finding (this study)
2784614	–	2785977	NUCmer repeat finding (this study)
2829020	–	2829928	NUCmer repeat finding (this study)
2828494	–	2829930	NUCmer repeat finding (this study)
2866469	–	2867121	NUCmer repeat finding (this study)
2867125	–	2867777	NUCmer repeat finding (this study)
2882593	–	2884330	NUCmer repeat finding (this study)
2944470	–	2945221	NUCmer repeat finding (this study)
2944354	–	2945226	NUCmer repeat finding (this study)
2971757	–	2971987	NUCmer repeat finding (this study)
2972105	–	2973463	NUCmer repeat finding (this study)
2973793	–	2975987	NUCmer repeat finding (this study)
2978623	–	2979060	NUCmer repeat finding (this study)
2982945	–	2983854	NUCmer repeat finding (this study)
2996010	–	2996104	NUCmer repeat finding (this study)
2996004	–	2996117	NUCmer repeat finding (this study)
2996011	–	2996155	NUCmer repeat finding (this study)
2996055	–	2996168	NUCmer repeat finding (this study)
3000741	–	3001893	NUCmer repeat finding (this study)
3002110	–	3003262	NUCmer repeat finding (this study)
3007038	–	3007167	NUCmer repeat finding (this study)
3007091	–	3007220	NUCmer repeat finding (this study)
3082466	–	3082654	NUCmer repeat finding (this study)
3100174	–	3100323	NUCmer repeat finding (this study)
3100175	–	3102010	NUCmer repeat finding (this study)
3100486	–	3102227	NUCmer repeat finding (this study)
3112884	–	3113179	NUCmer repeat finding (this study)
3113642	–	3113937	NUCmer repeat finding (this study)
3116388	–	3117192	NUCmer repeat finding (this study)
3119183	–	3120015	NUCmer repeat finding (this study)
3119333	–	3120103	NUCmer repeat finding (this study)
3119334	–	3120248	NUCmer repeat finding (this study)
3119938	–	3120464	NUCmer repeat finding (this study)

3119185	–	3120524	NUCmer repeat finding (this study)
3120523	–	3121879	NUCmer repeat finding (this study)
3120518	–	3121880	NUCmer repeat finding (this study)
3120521	–	3121881	NUCmer repeat finding (this study)
3120522	–	3121882	NUCmer repeat finding (this study)
3121934	–	3122406	NUCmer repeat finding (this study)
3122431	–	3122831	NUCmer repeat finding (this study)
3122012	–	3123208	NUCmer repeat finding (this study)
3122013	–	3123338	NUCmer repeat finding (this study)
3121937	–	3123354	NUCmer repeat finding (this study)
3121879	–	3123502	NUCmer repeat finding (this study)
3122230	–	3123561	NUCmer repeat finding (this study)
3123097	–	3123572	NUCmer repeat finding (this study)
3122011	–	3123576	NUCmer repeat finding (this study)
3122229	–	3123577	NUCmer repeat finding (this study)
3122436	–	3123581	NUCmer repeat finding (this study)
3132892	–	3133455	NUCmer repeat finding (this study)
3135788	–	3136351	NUCmer repeat finding (this study)
3155883	–	3156088	NUCmer repeat finding (this study)
3155937	–	3156089	NUCmer repeat finding (this study)
3171468	–	3171572	NUCmer repeat finding (this study)
3171463	–	3171607	NUCmer repeat finding (this study)
3171466	–	3171622	NUCmer repeat finding (this study)
3171522	–	3171626	NUCmer repeat finding (this study)
3171525	–	3171627	NUCmer repeat finding (this study)
3189482	–	3189567	NUCmer repeat finding (this study)
3189539	–	3189624	NUCmer repeat finding (this study)
3192195	–	3192300	NUCmer repeat finding (this study)
3192257	–	3192352	NUCmer repeat finding (this study)
3192248	–	3192353	NUCmer repeat finding (this study)
3192204	–	3192354	NUCmer repeat finding (this study)
3194138	–	3194451	NUCmer repeat finding (this study)
3194139	–	3195506	NUCmer repeat finding (this study)
3232651	–	3232810	NUCmer repeat finding (this study)
3232653	–	3232865	NUCmer repeat finding (this study)
3232707	–	3232866	NUCmer repeat finding (this study)
3239479	–	3239582	NUCmer repeat finding (this study)
3239471	–	3239743	NUCmer repeat finding (this study)
3239526	–	3239829	NUCmer repeat finding (this study)
3247062	–	3248167	NUCmer repeat finding (this study)
3252477	–	3253581	NUCmer repeat finding (this study)
3317534	–	3317739	NUCmer repeat finding (this study)
3319469	–	3319558	NUCmer repeat finding (this study)
3319570	–	3319657	NUCmer repeat finding (this study)
3334063	–	3334386	NUCmer repeat finding (this study)
3333758	–	3335596	NUCmer repeat finding (this study)
3336504	–	3336744	NUCmer repeat finding (this study)
3336563	–	3336802	NUCmer repeat finding (this study)
3351091	–	3351158	NUCmer repeat finding (this study)
3351147	–	3351214	NUCmer repeat finding (this study)
3376927	–	3377391	NUCmer repeat finding (this study)
3377649	–	3378269	NUCmer repeat finding (this study)
3378333	–	3378450	NUCmer repeat finding (this study)
3379364	–	3379828	NUCmer repeat finding (this study)
3380089	–	3380708	NUCmer repeat finding (this study)
3380911	–	3381026	NUCmer repeat finding (this study)
3381312	–	3382747	NUCmer repeat finding (this study)
3381313	–	3382749	NUCmer repeat finding (this study)

3481325	–	3482760	NUCmer repeat finding (this study)
3483946	–	3485111	NUCmer repeat finding (this study)
3528675	–	3529162	NUCmer repeat finding (this study)
3551225	–	3552584	NUCmer repeat finding (this study)
3551230	–	3552585	NUCmer repeat finding (this study)
3551227	–	3552587	NUCmer repeat finding (this study)
3552705	–	3554067	NUCmer repeat finding (this study)
3552713	–	3554068	NUCmer repeat finding (this study)
3552709	–	3554070	NUCmer repeat finding (this study)
3594263	–	3594402	NUCmer repeat finding (this study)
3594319	–	3594458	NUCmer repeat finding (this study)
3663699	–	3663825	NUCmer repeat finding (this study)
3663860	–	3663987	NUCmer repeat finding (this study)
3653792	–	3663995	NUCmer repeat finding (this study)
3710381	–	3711737	NUCmer repeat finding (this study)
3710380	–	3711738	NUCmer repeat finding (this study)
3710382	–	3712822	NUCmer repeat finding (this study)
3729364	–	3729681	NUCmer repeat finding (this study)
3730353	–	3731031	NUCmer repeat finding (this study)
3730351	–	3731064	NUCmer repeat finding (this study)
3732081	–	3732759	NUCmer repeat finding (this study)
3732079	–	3732792	NUCmer repeat finding (this study)
3735636	–	3736314	NUCmer repeat finding (this study)
3743198	–	3743501	NUCmer repeat finding (this study)
3743508	–	3743602	NUCmer repeat finding (this study)
3743711	–	3744009	NUCmer repeat finding (this study)
3743710	–	3744269	NUCmer repeat finding (this study)
3744460	–	3745336	NUCmer repeat finding (this study)
3744704	–	3745407	NUCmer repeat finding (this study)
3745500	–	3746006	NUCmer repeat finding (this study)
3746513	–	3747213	NUCmer repeat finding (this study)
3746540	–	3747233	NUCmer repeat finding (this study)
3746507	–	3747571	NUCmer repeat finding (this study)
3746616	–	3747586	NUCmer repeat finding (this study)
3746511	–	3747596	NUCmer repeat finding (this study)
3746303	–	3747763	NUCmer repeat finding (this study)
3748283	–	3748956	NUCmer repeat finding (this study)
3748322	–	3749015	NUCmer repeat finding (this study)
3748141	–	3749092	NUCmer repeat finding (this study)
3750167	–	3750773	NUCmer repeat finding (this study)
3750140	–	3750802	NUCmer repeat finding (this study)
3750137	–	3750862	NUCmer repeat finding (this study)
3750241	–	3751192	NUCmer repeat finding (this study)
3750134	–	3751201	NUCmer repeat finding (this study)
3751635	–	3753178	NUCmer repeat finding (this study)
3753331	–	3754361	NUCmer repeat finding (this study)
3755952	–	3756266	NUCmer repeat finding (this study)
3755951	–	3756510	NUCmer repeat finding (this study)
3756701	–	3757577	NUCmer repeat finding (this study)
3756933	–	3757609	NUCmer repeat finding (this study)
3757046	–	3757972	NUCmer repeat finding (this study)
3756942	–	3758031	NUCmer repeat finding (this study)
3757711	–	3758217	NUCmer repeat finding (this study)
3758734	–	3759401	NUCmer repeat finding (this study)
3758703	–	3759792	NUCmer repeat finding (this study)
3758496	–	3759959	NUCmer repeat finding (this study)
3760476	–	3761152	NUCmer repeat finding (this study)
3760516	–	3761183	NUCmer repeat finding (this study)

3760334	–	3761288	NUCmer repeat finding (this study)
3762386	–	3763337	NUCmer repeat finding (this study)
3762388	–	3763361	NUCmer repeat finding (this study)
3764037	–	3764678	NUCmer repeat finding (this study)
3764044	–	3765073	NUCmer repeat finding (this study)
3764043	–	3765086	NUCmer repeat finding (this study)
3765550	–	3767096	NUCmer repeat finding (this study)
3769514	–	3769853	NUCmer repeat finding (this study)
3770994	–	3771088	NUCmer repeat finding (this study)
3795044	–	3796412	NUCmer repeat finding (this study)
3795055	–	3796416	NUCmer repeat finding (this study)
3801958	–	3802230	NUCmer repeat finding (this study)
3802130	–	3802389	NUCmer repeat finding (this study)
3820393	–	3820473	NUCmer repeat finding (this study)
3820391	–	3820532	NUCmer repeat finding (this study)
3820448	–	3820589	NUCmer repeat finding (this study)
3841533	–	3842766	NUCmer repeat finding (this study)
3842149	–	3842904	NUCmer repeat finding (this study)
3842947	–	3843702	NUCmer repeat finding (this study)
3846461	–	3847692	NUCmer repeat finding (this study)
3847717	–	3847892	NUCmer repeat finding (this study)
3883550	–	3884921	NUCmer repeat finding (this study)
3890772	–	3892133	NUCmer repeat finding (this study)
3890777	–	3892135	NUCmer repeat finding (this study)
3890778	–	3892140	NUCmer repeat finding (this study)
3894406	–	3895519	NUCmer repeat finding (this study)
3930989	–	3932758	NUCmer repeat finding (this study)
3932294	–	3932794	NUCmer repeat finding (this study)
3932789	–	3933043	NUCmer repeat finding (this study)
3932676	–	3933045	NUCmer repeat finding (this study)
3933381	–	3933560	NUCmer repeat finding (this study)
3933402	–	3933562	NUCmer repeat finding (this study)
3933392	–	3934165	NUCmer repeat finding (this study)
3933995	–	3934768	NUCmer repeat finding (this study)
3934655	–	3934894	NUCmer repeat finding (this study)
3935229	–	3935411	NUCmer repeat finding (this study)
3935300	–	3935557	NUCmer repeat finding (this study)
3934871	–	3935806	NUCmer repeat finding (this study)
3935844	–	3936026	NUCmer repeat finding (this study)
3935915	–	3936172	NUCmer repeat finding (this study)
3935486	–	3936415	NUCmer repeat finding (this study)
3941225	–	3941626	NUCmer repeat finding (this study)
3941561	–	3941965	NUCmer repeat finding (this study)
3945098	–	3945597	NUCmer repeat finding (this study)
3946946	–	3947301	NUCmer repeat finding (this study)
3946945	–	3947491	NUCmer repeat finding (this study)
3945778	–	3947535	NUCmer repeat finding (this study)
3946934	–	3947652	NUCmer repeat finding (this study)
3947287	–	3947842	NUCmer repeat finding (this study)
3947276	–	3948003	NUCmer repeat finding (this study)
3947639	–	3948005	NUCmer repeat finding (this study)
3947990	–	3948082	NUCmer repeat finding (this study)
3947989	–	3948243	NUCmer repeat finding (this study)
3948218	–	3948399	NUCmer repeat finding (this study)
3947881	–	3948433	NUCmer repeat finding (this study)
3948013	–	3948507	NUCmer repeat finding (this study)
3948366	–	3948655	NUCmer repeat finding (this study)
3948610	–	3948727	NUCmer repeat finding (this study)

3948941	–	3949033	NUCmer repeat finding (this study)
3948312	–	3949401	NUCmer repeat finding (this study)
3949569	–	3949864	NUCmer repeat finding (this study)
3949750	–	3949872	NUCmer repeat finding (this study)
3948912	–	3950001	NUCmer repeat finding (this study)
3950830	–	3951329	NUCmer repeat finding (this study)
4039486	–	4040155	NUCmer repeat finding (this study)
4052971	–	4053438	NUCmer repeat finding (this study)
4053082	–	4053549	NUCmer repeat finding (this study)
4053634	–	4053900	NUCmer repeat finding (this study)
4053632	–	4053994	NUCmer repeat finding (this study)
4059984	–	4060591	NUCmer repeat finding (this study)
4078002	–	4078200	NUCmer repeat finding (this study)
4077999	–	4078201	NUCmer repeat finding (this study)
4087480	–	4087562	NUCmer repeat finding (this study)
4087537	–	4087619	NUCmer repeat finding (this study)
4120917	–	4120996	NUCmer repeat finding (this study)
4120974	–	4121053	NUCmer repeat finding (this study)
4155460	–	4155525	NUCmer repeat finding (this study)
4155517	–	4155582	NUCmer repeat finding (this study)
4156799	–	4156908	NUCmer repeat finding (this study)
4156858	–	4156967	NUCmer repeat finding (this study)
4215915	–	4216724	NUCmer repeat finding (this study)
4221089	–	4221937	NUCmer repeat finding (this study)
4222268	–	4222535	NUCmer repeat finding (this study)
4252882	–	4254332	NUCmer repeat finding (this study)
4265694	–	4266546	NUCmer repeat finding (this study)
4288136	–	4288256	NUCmer repeat finding (this study)
4301542	–	4301691	NUCmer repeat finding (this study)
4301685	–	4302008	NUCmer repeat finding (this study)
4301691	–	4303432	NUCmer repeat finding (this study)
4317966	–	4318157	NUCmer repeat finding (this study)
4318341	–	4319371	NUCmer repeat finding (this study)
4348725	–	4348832	NUCmer repeat finding (this study)
4353280	–	4353380	NUCmer repeat finding (this study)
4353331	–	4353431	NUCmer repeat finding (this study)
