# Supplementary material for: TGS-TB: Total Genotyping Solution for Mycobacterium tuberculosis Using Short-Read Whole-Genome Sequencing
Source: PLoS One. 2015 Nov 13;10(11):e0142951. doi: 10.1371/journal.pone.0142951 (PMC4643978; doi:10.1371/journal.pone.0142951)
Supplement: S3 Table — (PDF) [file pone.0142951.s009.pdf]

S3 Table. Loci information for short MIRU-VNTR

sMIRU-VNTR ID	Nucleotide position on H37Rv genome (NC_000962.3)	Alias	Repeat unit length (bp)
1	79505..79564	Mtub02 or H37Rv_0079_9	9
2	154112..154239	MIRU2 or H37Rv_0154_53	53
3	243135..243188	-	24
4	272855..272938	-	39
5	566273..566382	-	18
6	569812..569978	-	56
7	577286..577496	ETR-C or H37Rv_0577_58	58
8	595336..595449	-	58
9	917611..917735	-	58
10	960167..960323	MIRU10 or 0960c	53
11	1065356..1065417	-	30
12	1281897..1282021	QUB 1281c	60
13	1305495..1305661	-	62
14	1351196..1351282	-	27
15	1451779..1451995	QUB 1451 or H37Rv_1451_57	57
16	1612548..1612677	H37Rv_1612_21 or Qub23	21
17	1644249..1644367	MIRU16 or H37Rv_1644_53	53
18	1742790..1742848	-	30
19	1773949..1774114	-	15
20	1907457..1907578	-	56
21	2059430..2059600	MIRU20 or H37Rv_2059_77	77
22	2074433..2074636	Mtub24 or H37Rv_2074_56	56
23	2401817..2401927	Mtub30 or H37Rv_2401_58	58
24	2461320..2461531	ETR-B or H37Rv_2461_57	57
25	2575324..2575393	-	27
26	2703892..2704015	-	57
27	2778228..2778278	-	26
28	2827294..2827371	-	39
29	2861024..2861093	-	36
30	2990585..2990698	Mtub31 or H37Rv_2990_55	55
31	3007038..3007220	MIRU27 or Qub5	53
32	3171468..3171626	Mtub34 or H37Rv_3171_54	54
33	3189482..3189624	-	57
34	3290521..3290573	-	25
35	3316366..3316419	-	28
36	3510683..3510778	-	30
37	3843797..3843851	-	25
38	4087480..4087619	-	57
39	4155460..4155582	-	57
40	4160537..4160587	-	18
41	4348704..4348824	MIRU39 or H37Rv_4348_53	53
42	4373842..4373899	-	27
43	4393771..4393827	-	27

Composition of the different MIRU-VNTR sets:

Loci	Alias 1	Alias 2	24	15	12
154	MIRU02		X		X
424	Mtub04		X	X	
577	ETRC		X	X	
580	MIRU04	ETRD	X	X	X
802	MIRU40		X	X	X
960	MIRU10		X	X	X
1644	MIRU16		X	X	X
1955	Mtub21		X	X	
2059	MIRU20		X		X
2163b	QUB11b		X	X	
2165	ETRA		X	X	
2347	Mtub29		X		

Loci	Alias 1	Alias 2	24	15	12
2401	Mtub30		X	X	
2461	ETRB		X		
2531	MIRU23		X		X
2687	MIRU24		X		X
2996	MIRU26		X	X	X
3007	MIRU27	QUB5	X		X
3171	Mtub34		X		
3192	MIRU31	ETRE	X	X	X
3690	Mtub39		X	X	
4052	QUB26		X	X	
4156	QUB4156		X	X	
4348	MIRU39		X		X
